# Clinical Context Variables Collectively Rival Model Choice in Embedding-Based Retrieval: Multi-Corpus Benchmark Study

**DOI:** 10.2196/94241

**Published:** 2026-05-07

**Authors:** Yngve Mikkelsen

**Affiliations:** 1 Saïd Business School University of Oxford Oxford, England United Kingdom

**Keywords:** retrieval-augmented generation, clinical informatics, embedding models, benchmark, clinical documentation, BM25, dense retrieval

## Abstract

**Background:**

Retrieval-augmented generation (RAG) systems increasingly support clinical decision-making by grounding large language model outputs in verifiable evidence. The retrieval component is foundational: if the correct document is not retrieved, downstream generation cannot recover it. Despite this, embedding model selection for clinical RAG remains guided by general-domain benchmarks with limited clinical coverage. Given the heterogeneity of clinical documentation across institutions, specialties, and electronic health record systems, it is unclear whether general-domain model rankings generalize to clinical retrieval tasks.

**Objective:**

This study evaluated whether clinical context variables, corpus type (encompassing differences in document length, medical specialty, and structural characteristics), and query format have effects on retrieval performance comparable to or exceeding those of embedding model choice.

**Methods:**

Ten primary embedding models plus two ablation variants and a BM25 lexical baseline (13 retrieval configurations total) were benchmarked on three clinical corpora (MTSamples medical transcriptions, n=500; PMC-Patients case reports, n=500; Mistral-7B-generated synthetic clinical notes, n=500). Twelve embedding configurations were evaluated across 3 corpora × 2 query formats (keyword vs natural language) × 4 chunking strategies, yielding 294 experimental conditions. Primary metrics included MRR@10, P@1, Recall@10/20/50/100, and NDCG@10, with bootstrap confidence intervals. Relative factor contributions were quantified using factorial ANOVA with η² effect sizes, including all two-way interactions.

**Results:**

In a factorial ANOVA across 288 balanced embedding conditions, embedding model choice explained 40.8% of variance in MRR@10 (η²=0.408), corpus type 24.6%, and query format 19.2%. Chunking strategy explained minimal variance (η²=0.002). The model × query format interaction (η²=0.029, *P*<.001) indicated differential query sensitivity across models. A model × corpus interaction (η²=0.040, *P*<.001) indicated that model rankings shifted meaningfully across corpora. Combined context variables (corpus + query format + context interactions) explained 49.0% of total variance, compared with 47.6% for model-related effects. Model rankings were moderately unstable under keyword queries (Kendall τ=0.59, 95% CI [0.21, 0.89]) but highly stable under natural language queries (τ=0.82–0.87). BM25 achieved near-perfect retrieval on PMC-Patients in this known-item setting (MRR@10=0.999). Domain-specific models (BioBERT, ClinicalBERT) performed worse than general-purpose embeddings despite biomedical pretraining, with mean pairwise cosine similarity exceeding 0.90, indicating that all embeddings clustered in a narrow cone. A validation experiment using reduced-lexical-dependence queries—generated from GPT-4o-extracted metadata rather than document text—supported rank stability across query derivations (Kendall τ=0.59–0.90, mean 0.76, all *P*≤.004) and showed that BM25 remained strong on structured case reports (MRR@10=0.980).

**Conclusions:**

Clinical context variables explained as much variance in retrieval performance as embedding model choice, and model × corpus interactions showed that rankings are not portable across documentation types. Validation with reduced-lexical-dependence queries supported rank stability across query derivations. These results argue against reliance on general-domain leaderboards for clinical RAG deployment and support mandatory local validation as a methodological requirement.

## Introduction

### Background

Retrieval-augmented generation (RAG) has emerged as a leading strategy for grounding large language model (LLM) outputs in verifiable clinical knowledge, addressing persistent concerns about hallucinations and outdated training data [1,2]. In a typical clinical RAG pipeline, a user query is encoded by an embedding model, matched against a vector index of clinical documents, and the top-ranked passages are injected into an LLM prompt to guide answer generation. The retrieval component is foundational: if the correct document is not retrieved, no amount of generative sophistication can recover it.

Despite the centrality of the retrieval step, embedding model selection for clinical RAG remains largely guided by general-domain leaderboards such as the Massive Text Embedding Benchmark (MTEB) [3]. The implicit assumption is that models ranked highly on news articles, Wikipedia passages, and web queries will transfer effectively to clinical documentation. However, decades of health services research have demonstrated that clinical practice is profoundly heterogeneous. The Dartmouth Atlas project documented 4- to 10-fold variation in surgical procedure rates across hospital referral regions in the United States, with similar magnitudes observed internationally [4,5]. These variations are idiosyncratic and condition-specific rather than reflecting a general tendency toward aggressive or conservative care [5].

This practice heterogeneity directly affects clinical documentation. The structure and semantics of clinical notes vary widely across electronic health record (EHR) systems, sites, and institutions [6], as shown in a national analysis of over 215,000 ambulatory physicians [7]. Functional status documentation is context-specific, with variations driven by source instruments, information providers, practice settings, and institutions [8]. This heterogeneity poses a direct challenge to natural language processing model portability [6] and, by extension, to embedding model generalization in clinical retrieval.

Recent work on clinical RAG has made important progress. The Medical Information Retrieval-Augmented Generation Evaluation (MIRAGE) benchmark evaluated 41 combinations of corpora, retrievers, and backbone LLMs on 7663 medical questions and answers (QA), finding that corpus selection significantly affected performance and that no single configuration dominated [9]. A 2025 systematic review of 70 RAG-in-health care studies identified persistent challenges, including retrieval noise, domain shift, and limited evaluation frameworks [10]. Other studies have examined chunking strategies [11] and hallucination mitigation [12] for clinical RAG. However, existing benchmarks primarily evaluate retrieval via downstream QA accuracy on medical examination questions rather than through direct retrieval metrics on clinical documentation. To the author’s knowledge, no study has systematically benchmarked embedding models head-to-head on the heterogeneous clinical text that real-world RAG systems must process.

This paper addresses that gap with a controlled, multicorpus benchmark that isolates the retrieval component of clinical RAG. The study evaluates 10 embedding models (plus 2 ablation variants and Best Match 25 [BM25]) across 3 clinical corpora under 294 experimental conditions. The central hypothesis is that clinical context variables—primarily corpus type (encompassing differences in document length, specialty mix, and structural characteristics) and query format—produce effect sizes on retrieval performance comparable to or larger than those of embedding model choice. If confirmed, this would imply that local validation against institution-specific documentation is not merely a best practice but a methodological requirement for responsible clinical RAG deployment.

### Related Work

#### Clinical Practice and Documentation Heterogeneity

The observation that medical practice varies substantially across geographies and institutions is among the most robust findings in health services research. Wennberg and Gittelsohn’s [13] foundational small-area analysis in Vermont revealed large variations in hospitalizations, surgical procedures, and expenditures across hospital service areas that could not be explained by differences in population health needs. Dartmouth Atlas Project subsequently documented that these patterns persist nationally, with surgical procedure rates varying 4- to 8-fold across 306 hospital referral regions [4,5]. International comparisons showed that although absolute rates differ across countries, the relative degree of within-country variation is remarkably consistent, suggesting that clinical decision-making paradigms rather than environmental factors drive the phenomenon [5].

This variation in practice leads to corresponding variation in documentation. A study of EHR migration at 2 Mayo Clinic sites found that clinical note structure and semantics varied considerably across EHR implementations, with significant effects on natural language processing model portability [6]. Six distinct note composition strategies were identified nationally, with differential prevalence across specialties [7]. Functional status documentation was shown to be context-specific, with variations driven by source instruments, care settings, and institutional culture [8]. Together, these findings establish that clinical text available for RAG indexing reflects local practices, templates, and documentation cultures that differ markedly across institutions.

#### Embedding Models for Biomedical Text

Biomedical text embeddings have evolved through several generations. Domain-specific encoders, such as Biomedical Bidirectional Encoder Representations from Transformers (BioBERT) [14] and ClinicalBERT [15], applied continued pretraining of Bidirectional Encoder Representations from Transformers (BERT) on PubMed and Medical Information Mart for Intensive Care III (MIMIC-III) clinical notes. Purpose-built biomedical retrievers, including Biomedical Learning of Ontological Representations from Definitions (BioLORD) [16] and Medical Contrastive Pre-trained Transformer (MedCPT) [17], were trained with contrastive objectives on biomedical literature. General-purpose embedding models, such as BAAI (Beijing Academy of Artificial Intelligence) General Embedding (BGE) [18] (part of the FlagEmbedding family described in [19]), General Text Embeddings (GTE) [20], and Nomic Embed [21], have achieved strong MTEB performance. Recent LLM encoders (E5-Mistral-7B [22]) and commercial application programming interfaces (APIs; OpenAI text-embedding-3-small [23], hereafter OpenAI-emb3-small) have further expanded the landscape. MTEB [3] provides standardized comparisons, but its clinical coverage is limited to general biomedical text rather than institutional documentation.

#### RAG Evaluation in Health Care

MIRAGE [9] introduced the first systematic benchmark for medical RAG, evaluating RAG across 5 medical QA datasets. Its key finding—that corpus choice significantly affects downstream accuracy—is consistent with the present hypothesis. However, MIRAGE evaluates end-to-end QA accuracy on examination-style questions, conflating retrieval and generation quality. A 2025 evaluation of RAG variants for clinical decision support tested 12 pipeline configurations on 250 patient vignettes [12]. Studies on chunking for clinical RAG have demonstrated that adaptive strategies can improve precision [11]. This work complements these studies by isolating the retrieval component and systematically varying the clinical context rather than the pipeline architecture.

## Methods

### Corpora

Three corpora were selected to represent distinct clinical documentation contexts.

MTSamples (n=500) comprises deidentified medical transcription samples spanning 40 clinical specialties [24]. The full MTSamples dataset contains approximately 5000 documents; 500 were randomly sampled and stratified by specialty to preserve the original distribution. Documents shorter than 50 tokens or exact duplicates were excluded before sampling. The corpus includes operative reports, consultation notes, discharge summaries, and history-and-physical examinations, representing real-world dictated clinical documentation with varied formatting and narrative structures. The median document length was 391 (IQR 297-509) tokens.

PubMed Central (PMC)-Patients (n=500) comprises patient case descriptions from the PMC-Patients dataset [25], which aggregates structured case reports from PubMed Central. The source dataset contains approximately 167,000 patient summaries. A random sample of 500 English-language patient summaries was drawn, excluding entries shorter than 50 tokens, duplicates, and entries without a primary diagnosis label. These documents follow standardized academic reporting formats with explicit section headers. The median document length was 397 (IQR 305-495) tokens.

Synthetic clinical notes (n=500) were generated using Mistral-7B-Instruct-v0.2 (mistralai/Mistral-7B-Instruct-v0.2) with structured prompts specifying the clinical specialty (see the “Use of Language Models for Synthetic Data Generation” section and Multimedia Appendix 1 for prompt templates). Each note was generated independently with temperature 0.8 and top_p 0.9 to encourage diversity. All outputs were manually screened to confirm the absence of real patient identifiers. The median document length was 421 (IQR 312-518) tokens.

To characterize the synthetic corpus relative to the other 2, I computed corpus-level lexical statistics on a 100-document subset (5 per specialty). The synthetic corpus exhibited high structural uniformity: document length SD of 26 tokens (vs SD 143 for MTSamples and SD 163 for PMC-Patients, as estimated from IQR), and a mean document-level type-token ratio of 0.597 (SD 0.048). Cross-specialty vocabulary overlap was high (mean pairwise Jaccard 0.266; range 0.179-0.434), with 71%-94% of each specialty’s vocabulary appearing in at least one other specialty (per-specialty counts: 350-502 shared of 420-545 total unique terms). Term-frequency entropy was 9.14 bits (of 11.44 maximum), indicating moderate lexical diversity but substantially less than would be expected from real institutional EHR data. The structural regularity of the synthetic corpus—reflected in identical section-based and 512-token chunk counts (Table 2)—likely contributes to its uniformly low retrieval performance and confirms that it functions more as a stress test of cross-specialty disambiguation than as a representative third clinical documentation context. A potential circularity concern—that Mistral-generated text might preferentially advantage architecturally related models—was tested by comparing model rank positions across corpora: E5-Mistral-7B-ablation maintained rank 8 across all 3 corpora, and Phi-3-mini dropped from rank 9 (MTSamples) to rank 11 (synthetic). No Mistral-related model showed a relative rank improvement on the synthetic corpus, indicating no evidence of circularity.

### Query Generation

#### Deterministic Generation of Keyword and Natural-Language Query Formats

For each document, 2 query types were generated using deterministic heuristics (no language model was used for query generation in the main benchmark). Keyword queries comprised 3-6 clinical terms extracted heuristically from each document by selecting capitalized medical terms and available metadata fields, such as specialty labels (eg, “Cardiology Dyspnea Hypertension HbA_1c_”), simulating search-box behavior in clinical information systems. Natural language queries consisted of the first 1-2 sentences of each document’s clinical narrative (eg, the opening of the History of Present Illness), representing a high-overlap retrieval scenario. For MTSamples, the description field was used when available. Both query types were derived directly from the target document text.

#### Known-Item Retrieval Design

As both query types were derived from target documents, the retrieval task constitutes known-item retrieval: queries are designed to match a specific known document. This design was chosen because it provides an unambiguous ground truth—each query has exactly 1 correct document—without requiring expert relevance judgments, which would be prohibitively expensive at the scale of 294 conditions. The trade-off is that absolute mean reciprocal rank (MRR) values are not directly comparable to production retrieval settings where queries are formulated independently; the primary analytical object is relative model rankings, which are interpretable under this design. This approach increases lexical overlap between queries and targets, which may benefit lexical methods such as BM25 and embedding models with strong lexical dependence. Using verbatim opening sentences as natural language queries and heuristically extracted terms as keyword queries produces particularly high overlap, establishing a controlled high-overlap retrieval scenario for lexical methods. However, heuristic term extraction also introduces lexical noise (eg, nondiagnostic capitalized terms), which can depress dense retrieval performance; the net effect on absolute scores is therefore not necessarily upward for all models (see the “Validation With Reduced-Lexical-Dependence Queries” section). The near-perfect BM25 performance on PMC-Patients (MRR@10=0.999 for natural language queries) reflects this design, as the opening sentences of structured case reports contain rare medical terms directly present in target documents, giving BM25 an extreme advantage through inverse document frequency weighting. Absolute performance values are therefore not directly representative of production retrieval; in settings where user queries are formulated independently of target documents, performance would differ for all models. Relative comparisons across models and corpora remain valid within this design, as all conditions share the same query derivation method. A validation experiment using LLM-generated reduced-lexical-dependence queries supported the stability of model rankings across query derivations (see the “Validation With Reduced-Lexical-Dependence Queries” section).

### Models

Ten embedding models, plus BM25, were evaluated across 6 architectural categories (Table 1). All models were evaluated using vendor-recommended pooling strategies and task-specific prefixes, as detailed in Multimedia Appendix 2. Two ablation variants were also included: Nomic-embed-text without its search_query prefix, and E5-Mistral-7B using mean pooling instead of the vendor-specified last-token (end of sequence [EOS]) pooling, yielding 12 embedding configurations and 13 total retrieval configurations, including BM25.

**Table 1:** Model configurations and architectural categories.

Model	Category	Parameters	Dimensionality	Source/notes
BM25^a^	Lexical baseline	N/A^b^	N/A	Okapi BM25 (k1=1.5, b=0.75)
BioBERT^c^	Domain encoder	110 million	768	dmis-lab/biobert-v1.1
ClinicalBERT	Domain encoder	110 million	768	medicalai/ClinicalBERT
BioLORD^d^-2023	Biomedical retriever	110 million	768	FremyCompany/BioLORD-2023
MedCPT^e^	Biomedical retriever	110 million	768	ncbi/MedCPT-Query/Article-Encoder^f^
BGE^g^-base-en-v1.5	General embedding	110 million	768	Beijing Academy of Artificial Intelligence/bge-base-en-v1.5
GTE^h^-base	General embedding	137 million	768	thenlper/gte-base
Nomic-embed-v1.5	General embedding	137 million	768	nomic-ai/nomic-embed-text-v1.5
OpenAI-emb3-small	General application programming interface	N/A	1536	Application programming interface (cl100k_base tokenizer)
E5-Mistral-7B	General large language model	7 billion	4096	intfloat/e5-mistral-7b-instruct
Phi-3-mini-128k	General large language model	3.8 billion	3072	microsoft/Phi-3-mini-128k-instruct

^a^BM25: Best Match 25.

^b^N/A: not applicable.

^c^BioBERT: Biomedical Bidirectional Encoder Representations from Transformers.

^d^BioLORD: Biomedical Learning of Ontological Representations from Definitions.

^e^MedCPT: Medical Contrastive Pre-trained Transformer.

^f^MedCPT uses a dual-encoder architecture with separate query (ncbi/MedCPT-Query-Encoder) and article (ncbi/MedCPT-Article-Encoder) encoders. Performance reflects this asymmetric design rather than a single shared representation space.

^g^BGE: BAAI General Embedding.

^h^GTE: General Text Embeddings.

### Chunking Strategies

#### Comparison of Chunking Strategies and Tokenization Standardization

Each corpus was indexed using 4 chunking strategies: (1) the full document as a single vector; (2) section-based splitting at detected clinical section headers (eg, “History of Present Illness,” “Assessment/Plan”); (3) fixed 512-token nonoverlapping chunks that respect sentence boundaries; and (4) fixed 256-token nonoverlapping chunks that respect sentence boundaries. Token counts were computed with the cl100k_base tokenizer (tiktoken library, version 0.5.1). This tokenizer was chosen for its stability and widespread adoption, providing consistent approximate token budgeting across conditions; word count–based sizing would not account for subword segmentation differences, while model-specific tokenizers would confound chunking with model-dependent tokenization. The embedding models’ own tokenizers handle input encoding independently. Table 2 shows the mean number of index items per chunking strategy and corpus.

**Table 2:** Mean number of index items per chunking strategy and corpus. The identical section-based and 512-token counts for the synthetic corpus (987) reflect the similar document structure: Mistral-7B–generated notes average 421 tokens with consistent section boundaries, so section-based and 512-token splitting produce nearly identical break points.

Corpus	Full, n	Section, n	512-token splitting, n	256-token splitting, n
MTSamples	500	500	1067	2158
PMC^a^-Patients	500	501	987	1960
Synthetic	500	987	987	1499

^a^PMC: PubMed Central.

#### Chunking Ground Truth

For chunked conditions, a query’s target document was considered successfully retrieved if any chunk from that document appeared in the top-k results (document-level evaluation). As chunking increases the total number of index items (Table 2), chance-level retrieval performance varies across chunking conditions, although the effect is small at k=10 relative to minimum index sizes of 500.

### Evaluation Metrics

#### Primary Retrieval Metrics and Bootstrap CIs

Primary retrieval metrics included mean reciprocal rank at cutoff 10 (MRR@10) as the primary performance measure, precision at 1 (P@1), recall at 10/20/50/100, and normalized discounted cumulative gain at 10 (NDCG@10). Bootstrap 95% CIs (1000 resamples, percentile method) were computed for MRR@10.

Supplementary analyses included (1) document-length sensitivity, with documents binned into terciles; (2) lexical overlap analysis measuring Spearman correlation between query-document Jaccard similarity (computed on lowercased, punctuation-stripped tokens with English stop words removed) and retrieval rank; (3) embedding geometry diagnostics; and (4) factorial ANOVA with **η^2^** effect sizes.

#### Embedding Geometry Metrics

Anisotropy was computed as the mean pairwise cosine similarity across 1000 randomly sampled embedding pairs; values approaching 1.0 indicate that all embeddings point in approximately the same direction, making cosine similarity nondiscriminative [26]. Average self-similarity is the mean cosine similarity of each document embedding to all others; high values (>0.95) indicate near-complete loss of retrieval capacity. Effective rank was computed as the exponential of the Shannon entropy of the normalized singular value spectrum of the embedding matrix, measuring the effective dimensionality used by the model [27]. First principal component variance ratio was computed as the proportion of total variance explained by the first principal component of the embedding matrix; higher values indicate more concentrated, less isotropic embedding distributions.

### Experimental Procedure

All experiments were run on a single NVIDIA H100 80 GB graphical processing unit (GPU). The 12 embedding configurations were evaluated across 3 corpora × 2 query formats × 4 chunking strategies = 288 conditions. BM25 was evaluated across 3 corpora × 2 query formats × 1 (full-document only) = 6 conditions; it was restricted to full-document indexing because chunk-level BM25 retrieval introduces a passage-to-document aggregation step absent from the embedding pipeline, and because full-document BM25 represents the standard baseline in information retrieval benchmarks. Passage-level BM25 with score aggregation (eg, MaxP, SumP) is a common alternative that may interact with chunking differently than dense retrieval; this is left for future work. The total of 294 conditions reflects the complete factorial for embedding models plus the BM25 subset. For each condition, cosine similarity (or BM25 scores) was computed between query and document/chunk representations, items were ranked, and results were evaluated against the known single relevant document per query. BM25 was implemented using rank_bm25 (version 0.2.2) with default parameters (k1=1.5, b=0.75). A post hoc sensitivity analysis over k1 ∈ {1.0, 1.2, 1.5, 2.0} and b ∈ {0.25, 0.5, 0.75, 1.0} (16 parameter combinations) confirmed that BM25 performance was robust to parameter choice: the maximum MRR@10 spread across all parameter combinations was 0.038 (PMC-Patients, keyword), 0.032 (MTSamples, keyword), and 0.011 (synthetic, keyword)—far smaller than cross-model differences. Default parameters (k1=1.5, b=0.75) fell within 0.014 of the best-performing combination in all conditions, using the same lowercased, punctuation-stripped tokenization as for lexical overlap analysis. Models were loaded sequentially, with explicit GPU memory clearing between evaluations.

Two design constraints are relevant to interpretation. First, the single-relevant-document assumption, while standard in known-item retrieval benchmarking, differs from production settings where multiple documents may be relevant to a given query; this may differentially affect model rankings. Second, the corpora of 500 documents each are smaller than production indices; scaling effects on retrieval difficulty are not captured.

### Use of Language Models for Synthetic Data Generation

Mistral-7B-Instruct-v0.2 (mistralai/Mistral-7B-Instruct-v0.2) was used to generate 500 synthetic clinical notes. The model was loaded in half-precision (float16) on a single H100 GPU. Each note was generated in an independent inference call with temperature 0.8 and top_p 0.9, using a structured prompt that specified the clinical specialty (drawn from 20 specialties; see Multimedia Appendix 1 for the prompt template). No conversation history was retained between generations to prevent cross-contamination. Notes were screened for inadvertent real patient identifiers (none were found).

For the main benchmark, query generation relied on deterministic heuristics rather than language models. Keyword queries were constructed by extracting capitalized medical terms and available metadata fields (eg, specialty labels) from each document. Natural language queries consisted of the first 1-2 sentences of each document’s clinical narrative, or the description field for MTSamples when available. This approach produces high lexical overlap between queries and target documents, creating a high-overlap retrieval scenario (see the “Query Generation” section). No language model was used for query generation in the primary evaluation.

GPT-4o (gpt-4o-2024-05-13, OpenAI) was used exclusively in the validation experiment (see the “Validation With Reduced-Lexical-Dependence Queries” section) for metadata extraction (temperature 0.0) and for metadata-only query generation (temperature 0.3). No language model was used for manuscript drafting, analysis, or interpretation of results.

### Statistical Analysis

To quantify the relative contribution of each experimental factor to retrieval performance, a type II factorial ANOVA was performed on MRR@10 with fixed effects for model, corpus, query format, and chunking strategy, plus all 2-way interactions: model × corpus, model × query format, corpus × query format, corpus × chunking, model × chunking, and query format × chunking. Effect sizes were reported as η^2^ (sum of squares for each factor divided by the total sum of squares). The primary analysis used the 288 balanced embedding model conditions (excluding BM25, which lacked chunking conditions). A sensitivity analysis including BM25 (full-document conditions only; N=78) was conducted to assess robustness. A secondary analysis replaced individual model identity (12 levels) with architectural category (6 levels: domain encoder, biomedical retriever, general embedding, general API, general LLM, and ablation) to distinguish between model choice and architectural category as explanatory factors.

This ANOVA was performed on condition-level aggregated MRR@10 values (N=288 observations) rather than on per-query reciprocal ranks. This design treats the ANOVA as a descriptive variance decomposition across experimental settings rather than a formal inferential test. As the same documents and queries contribute to all model conditions, the observations are not independent, and *P* values should be interpreted as indicators of relative factor importance rather than as classical hypothesis tests. *P* values are reported alongside η^2^ for completeness, but effect sizes are emphasized as the primary basis for interpretation. A bootstrap sensitivity analysis (resampling conditions with replacement, 1000 iterations) confirmed that the η^2^ decomposition is stable: embedding model 95% CIs (percentile) 0.392-0.522; corpus 95% CIs (percentile) 0.183-0.289; query format 95% CIs (percentile) 0.129-0.216; and chunking 95% CIs (percentile) 0.000-0.005. Model ranking stability was assessed using Kendall τ with bootstrap 95% CIs (10,000 resamples) and Spearman ρ. All analyses were conducted in Python 3.11 (Python Foundation) using statsmodels 0.14, scipy 1.12, and numpy 1.26.

### Ethical Considerations

This study used only publicly available or synthetically generated datasets and did not involve human research. MTSamples consists of publicly posted, deidentified medical transcriptions accessed in accordance with the site’s terms of service [24]. PMC-Patients includes previously published case reports from PubMed Central. Synthetic clinical notes were generated using Mistral-7B-Instruct-v0.2 and contain no real patient data. As no individually identifiable patient information was processed, institutional review board approval and informed consent were not required, and no participant compensation was applicable. All data sources are publicly accessible; no privacy-restricted data were generated, accessed, or stored.

## Results

### Variance Decomposition: Context Variables Collectively Match Model Choice Effects

Table 3 presents the factorial ANOVA decomposition of MRR@10 across all 288 balanced embedding conditions, including all 2-way interactions. Embedding model choice was the largest single factor (η^2^=0.408, 40.8% of variance), followed by corpus (η^2^=0.246, 24.6%) and query format (η^2^=0.192, 19.2%). Model × query format was notable (η^2^=0.029, *P*<.001), indicating that models differ in their sensitivity to query type. Chunking strategy contributed little to the variance decomposition, both as a main effect (η^2^=0.002, *P*=.009) and in most interactions: corpus × chunking (η^2^=0.001, *P*=.41) and model × chunking (η^2^=0.002, *P*=.99, reflecting near-zero between-cell variance across 33 degrees of freedom). Query format × chunking was small but detectable (η^2^=0.002, *P*=.003). Interactions were also observed for model × corpus (η^2^=0.040, *P*<.001) and corpus × query format (η^2^=0.052, *P*<.001). The combined model explained 97.4% of the variance (*R*^2^=0.974).

**Table 3:** Factorial ANOVA of MRR@10^a^ across 288 embedding conditions (all 2-way interactions).

Factor	*η* ^2b^	*F* test^c^ (*df*)	*P* value^c^
Embedding model	0.408	269.72 (11, 193)	<.001
Corpus	0.246	895.22 (2, 193)	<.001
Query format	0.192	1399.11 (1, 193)	<.001
Chunking strategy	0.002	3.99 (3, 193)	.009
Model × corpus	0.040	13.17 (22, 193)	<.001
Model × query format	0.029	18.84 (11, 193)	<.001
Corpus × query format	0.052	187.62 (2, 193)	<.001
Corpus × chunking	0.001	1.03 (6, 193)	.41
Model × chunking	0.002	0.52 (33, 193)	.99
Query × chunking	0.002	4.88 (3, 193)	.003
Residual	0.027	N/A^d^	N/A

^a^MRR@10: mean reciprocal rank at 10.

^b^η^2^ is calculated as the sum of squares (factor)/sum of squares (total). Here, N=288 (12 embedding models × 3 corpora × 2 query formats × 4 chunking strategies).

^c^*F* and *P* values are not defined for the “Residual” row. The value 193 (*df*_numerator_) for that row is the residual degrees of freedom used as *df*_denominator_ by every *F* test in the table.

^d^N/A: not applicable.

Context variables collectively explain as much variance as model choice: corpus + query format + corpus × query format=49.0% versus model + model × corpus + model × query format=47.6%. While model choice is the single largest factor, optimizing model selection while ignoring corpus characteristics and query design leaves approximately half of the available performance variation unaddressed. The model × corpus interaction (*F*_22,193_=13.17, *P*<.001) indicates that model rankings shift meaningfully across corpora—not merely as additive offsets but as rank reordering. The model × query format interaction (*F*_11,193_=18.84, *P*<.001) further shows that models differ in their sensitivity to query type, meaning that query reformulation affects models unequally.

A secondary analysis replacing individual model identities with an architectural category (6 levels) showed that the category explained 37.2% of the variance—lower than the 40.8% explained by individual models, with the 3.6% difference reflecting within-category model variation. Context variables explained an even larger relative share than the architectural category. A sensitivity analysis restricted to full-document conditions, including BM25 (N=78), yielded a consistent pattern: model 46.8%, corpus 24.8%, query format 16.4% (*R*^2^=0.880).

These results were robust to metric choice. Kendall τ between model rankings under MRR@10 and P@1 averaged 0.985 across the 6 dataset × query format conditions (12 models each), indicating near-identical rankings. Concordance with recall@10 was also high (τ=0.894) and with recall@50 somewhat lower (τ=0.822), as expected given the broader retrieval window. NDCG@10 correlated perfectly with MRR@10 (τ=0.985), which is expected when each query has exactly 1 relevant document—in this setting, the 2 metrics are monotone transforms of each other. NDCG@10 is therefore omitted from the primary results and retained only in Multimedia Appendix 3.

To address the nonindependence limitation of the condition-level ANOVA, I fitted a linear mixed-effects model to 39,000 per-query reciprocal ranks (13 models × 3 corpora × 2 query formats × ≈500 queries) with query_id as a random intercept. All 3 fixed effects were significant by likelihood ratio tests (model: *χ*^2^_12_=13,540.6, *P*<.001; dataset: *χ*^2^_2_=801.6, *P*<.001; query format: *χ*^2^_1_=4456.9, *P*<.001). The intraclass correlation coefficient was 0.210, indicating that 21.0% of residual variance was attributable to between-query difficulty—a source that the condition-level ANOVA absorbs into the residual. Despite this reattribution, the relative importance ordering was preserved: model remained the largest contributor, followed by dataset and query format. Fixed effects explained 37.3% of total variance (pseudo-*R*^2^=0.373). These results confirm that the ANOVA findings are robust to the nonindependence concern. The condition-level *R*^2^=0.974 is partly inflated by aggregation over heterogeneous queries; the per-query pseudo-*R*^2^=0.373 provides a more conservative estimate of variance attributable to experimental factors.

### Model Rankings Are Corpus-Dependent

Table 4 presents MRR@10 for all retrieval configurations under keyword queries with full-document indexing. Model rankings were moderately unstable across corpora. Nomic-embed-text achieved the highest MRR@10 on MTSamples (0.768, 95% CI 0.734-0.800) but fell to 0.460 on PMC-Patients, where BM25 dominated (0.881, 95% CI 0.853-0.906)—a margin of +0.312 over the best embedding model (MedCPT; 0.569).

**Table 4:** MRR@10^a^ by model and corpus (keyword queries, full-document indexing)^b,c^.

Model	Category	MTSamples	PMC^d^-Patients	Synthetic
Nomic-embed-text	General embedding	*0.768*	0.460	*0.288*
BGE^e^-base-en-v1.5	General embedding	0.759	0.459	0.253
GTE^f^-base	General embedding	0.730	0.413	0.219
OpenAI-emb3-small	General application programming interface	0.711	0.410	0.273
BM25^g^	Lexical	0.694	*0.881*	0.266
MedCPT^h^	Biomedical retriever	0.624	0.569	0.212
BioLORD^i^-2023	Biomedical retriever	0.581	0.225	0.162
E5-Mistral-7B (mean-pooling, ablation)	General large language model	0.344	0.195	0.152
Phi-3-mini	General large language model	0.175	0.140	0.031
BioBERT^j^	Domain encoder	0.165	0.148	0.040
ClinicalBERT	Domain encoder	0.129	0.046	0.014
E5-Mistral-7B (end of sequence, vendor)	General v	0.062	0.169	0.042
Nomic-embed-text (no prefix, ablation)	Ablation	0.746	0.399	0.314

^a^MRR@10: mean reciprocal rank at 10.

^b^Ablation variants are labeled with their pooling or prefix configuration in parentheses.

^c^Italics indicates best in column.

^d^PMC: PubMed Central.

^e^BGE: BAAI General Embedding.

^f^GTE: General Text Embeddings.

^g^BM25: Best Match 25.

^h^MedCPT: Medical Contrastive Pre-trained Transformer.

^i^BioLORD: Biomedical Learning of Ontological Representations from Definitions.

^j^BioBERT: Biomedical Bidirectional Encoder Representations from Transformers.

Rank stability varied by query type (Table 5). For keyword queries, Kendall τ between MTSamples and PMC-Patients was 0.590 (95% CI 0.211-0.889), indicating a moderate positive association with substantial reordering among individual models. For natural language queries, rankings were much more stable (τ=0.821-0.872 across all corpus pairs). This suggests that keyword-based retrieval is more sensitive to corpus-specific vocabulary, whereas natural language queries allow models to exploit semantic similarity more consistently.

**Table 5:** Rank stability across corpora (Kendall τ with bootstrap 95% CI).

Corpus pair	Spearman ρ	Kendall τ (95% CI)	Query type
MTSamples vs PMC^a^-Patients	0.753	0.590 (0.211-0.889)	Keyword
MTSamples vs synthetic	0.890	0.744 (0.472-0.944)	Keyword
PMC-Patients vs synthetic	0.775	0.641 (0.127-0.971)	Keyword
MTSamples vs PMC-Patients	0.934	0.821 (0.531-1.000)	Natural language
MTSamples vs synthetic	0.940	0.846 (0.559-1.000)	Natural language
PMC-Patients vs synthetic	0.962	0.872 (0.671-1.000)	Natural language

^a^PMC: PubMed Central.

### Query Format Effect

Table 6 presents the MRR@10 difference when switching from keyword to natural language queries. The effect was positive for all but 2 model-corpus combinations (MedCPT on synthetic: Δ=−0.011 and E5-Mistral-7B on synthetic: Δ=−0.001) and often exceeded the gap between the best and worst models. On PMC-Patients, BioLORD-2023 improved from 0.225 to 0.884 (Δ=+0.659), a nearly 4-fold improvement. This single-variable change exceeded the entire range of model performance under keyword queries (0.835 range). On MTSamples, the average query format effect was +0.171 MRR@10 points; on PMC-Patients, it was +0.399. The MedCPT exception is consistent with its contrastive training on structured PubMed query-article pairs that resemble keyword queries; natural language reformulation may disrupt the learned query-document alignment for this model.

**Table 6:** Query format effect: MRR@10^a^ difference (natural language minus keyword), full-document indexing. Seven models were selected to represent each architectural category and the widest effect range; full results are presented in Multimedia Appendix 3.

Model	Δ MTSamples	Δ PMC^b^-Patients	Δ synthetic
BioLORD^c^-2023	+0.188	+0.659	+0.257
GTE^d^-base	+0.156	+0.489	+0.250
Nomic-embed-text	+0.121	+0.490	+0.221
BGE^e^-base-en-v1.5	+0.123	+0.424	+0.241
BM25^f^	+0.189	+0.118	+0.364
OpenAI-emb3-small	+0.151	+0.419	+0.181
MedCPT^g^	+0.101	+0.151	−0.011

^a^MRR@10: mean reciprocal rank at 10.

^b^PMC: PubMed Central.

^c^BioLORD: Biomedical Learning of Ontological Representations from Definitions.

^d^GTE: General Text Embeddings.

^e^BGE: BAAI General Embedding.

^f^BM25: Best Match 25.

^g^MedCPT: Medical Contrastive Pre-trained Transformer.

### Document Length Introduces Systematic Bias

Retrieval performance degraded with document length across most models. On PMC-Patients, OpenAI-emb3-small showed the largest bias: MRR@10 of 0.515 for short documents versus 0.282 for long (Δ=+0.233). MedCPT was the sole exception, showing stable or slightly improved performance on longer documents (short 0.548 vs long 0.599, Δ=−0.051 on PMC-Patients), likely attributable to contrastive training on longer PubMed articles. The length bias was corpus-dependent: on the synthetic corpus, several models, including Nomic-embed-text and BGE-base-en-v1.5, showed reversed or negligible length effects.

The length-tercile analysis serves as a partial proxy for specialty-level variation, because clinical specialties differ systematically in document length and terminology. However, the condition-level ANOVA uses corpus type as a single factor rather than modeling specialty individually. Within MTSamples (40 specialties) and the synthetic corpus (20 specialties, 5 documents each), specialty-specific retrieval difficulty likely varies: surgical operative reports use distinctive procedural vocabulary that may be easier to retrieve than general medicine notes with overlapping symptom terms. The length-tercile spread (up to Δ=0.233 for OpenAI-emb3-small on PMC-Patients) suggests that within-corpus document characteristics meaningfully affect retrieval, and specialty is a plausible driver of this variation. A per-query stratified analysis by specialty was not conducted because the condition-level design aggregates across documents; future work with per-query retrieval logs could decompose the corpus effect into specialty-level components.

### Lexical Overlap Correlates With Retrieval Success

Spearman correlations between query-document Jaccard similarity and retrieval rank were negative across nearly all models (higher overlap=better rank). BM25 showed the strongest correlation on MTSamples (ρ=−0.556). Among embedding models, E5-Mistral-7B-ablation showed strong lexical dependence (ρ=−0.498), while MedCPT showed near-zero correlation on PMC-Patients (ρ=−0.008), indicating genuinely semantic retrieval. On synthetic, BioBERT and ClinicalBERT showed positive (reversed) correlations (ρ=+0.105 and +0.082), indicating that retrieval was effectively random with respect to lexical content—consistent with the degenerate embedding geometry observed for these models (see the “Domain-Specific Pretraining Does Not Guarantee Retrieval Quality” section).

### Domain-Specific Pretraining Does Not Guarantee Retrieval Quality

BioBERT and ClinicalBERT ranked 11th and 12th among 13 configurations across all corpora despite biomedical pretraining. Embedding geometry analysis revealed the mechanism: both exhibited anisotropy exceeding 0.90 (mean pairwise cosine similarity over 1000 random pairs; see the “Evaluation Metrics” section) across all corpora (Table 7), indicating that embeddings are dominated by a narrow cone in which cosine similarity between arbitrary document pairs is uniformly high. Self-similarity scores of 0.97-0.99 confirmed a near-complete loss of discriminative capacity. By contrast, BioLORD-2023 (contrastive training, same domain) achieved anisotropy of only 0.25-0.40 and 3-5× better retrieval performance, demonstrating that the training objective matters more than the pretraining domain.

**Table 7:** Embedding geometry and retrieval performance (MTSamples, keyword, and full-document).

Model	Anisotropy	Self-similarity	Effective rank	MRR@10^a^	Category
ClinicalBERT	0.905	0.969	222	0.129	Domain encoder
BioBERT^b^	0.950	0.982	257	0.165	Domain encoder
Phi-3-mini	0.974	0.993	286	0.175	General large language model
BioLORD^c^-2023	0.248	0.681	267	0.581	Biomedical retriever
BGE^d^-base-en-v1.5	0.671	0.842	314	0.759	General embedding
Nomic-embed-text	0.652	0.847	302	0.768	General embedding
OpenAI-emb3-small	0.462	0.789	319	0.711	General application programming interface

^a^MRR@10: mean reciprocal rank at 10.

^b^BioBERT: Biomedical Bidirectional Encoder Representations from Transformers.

^c^BioLORD: Biomedical Learning of Ontological Representations from Definitions.

^d^BGE: BAAI General Embedding.

### LLM-Based Encoders: Pooling Strategy Is Critical

E5-Mistral-7B with the vendor-specified last-token (EOS) pooling ranked last across all 13 configurations on every corpus, achieving its lowest single-condition MRR@10 of 0.062 (MTSamples, full-document, and keyword). The E5-Mistral-7B model card recommends last-token pooling with query instructions for optimal performance; the finding that this configuration fails on clinical text—even when following vendor guidance—is consistent with a task-distribution misalignment between the model’s training data and clinical documentation. A mean-pooling ablation improved performance 5.5× to 0.344, suggesting that clinical text does not produce the token-position patterns EOS pooling was optimized for. Phi-3-mini (3.8 billion parameters, mean pooling) scored only 0.175, failing to outperform 110-million-parameter general embedding models. Larger parameter counts do not compensate for misaligned training objectives.

### Chunking Strategy Effects Are Modest

Fixed 256-token chunking achieved the highest mean MRR@10 across embedding conditions on 2 of 3 corpora: 0.580 (MTSamples) and 0.543 (PMC-Patients), compared with 0.569 and 0.502 for full-document indexing. On the synthetic corpus, full-document and fixed-256 were tied at 0.238. The maximum chunking effect across corpora was Δ=0.066 (between fixed-256 and fixed-512 on PMC-Patients)—small in variance-decomposition terms (η^2^=0.002) but potentially meaningful for applied retrieval, where even modest MRR gains affect user experience. To contextualize the impact of chunking, under keyword queries, the largest MRR@10 difference between chunking strategies within a single model was 0.106 (PMC-Patients), whereas the cross-model difference within a single corpus reached 0.671 (MTSamples). Across all 3 corpora, the maximum chunking effect ranged from 11% to 20% of the corresponding corpus-specific model effect (defined as within-model chunk spread divided by cross-model MRR@10 spread, restricted to keyword queries: MTSamples 0.077/0.671=11.5%; PMC-Patients 0.106/0.527=20.1%; and synthetic 0.047/0.288=16.3%). Practitioners should therefore prioritize model selection over chunking strategy, selecting the latter based on application constraints (latency and context window size) rather than retrieval performance alone. Chunking had small effects in most ANOVA interactions (corpus × chunking, *P*=.41, model × chunking, *P*=.99). Query × chunking was small but detectable (*P*=.003), suggesting that chunking effects may differ slightly between keyword and natural language queries. These chunking results apply to the dense retrieval pipelines evaluated here; lexical retrieval may behave differently under chunking. Chunking may also become more important at larger scales, where documents routinely exceed model context windows.

### Validation With Reduced-Lexical-Dependence Queries

To test whether relative model rankings are artifacts of the known-item retrieval design, the evaluation was repeated using metadata-only queries for a random subset of 100 documents per corpus (300 total). For each document, GPT-4o (gpt-4o-2024-05-13, temperature 0.0) extracted structured metadata—specialty, note type, primary diagnosis, secondary diagnoses, and patient demographics—from the document text. Queries were then generated by GPT-4o (temperature 0.3) from these metadata fields alone, without access to the document text. Although the metadata extraction step reads the document, the resulting metadata representation substantially reduces lexical dependence: mean Jaccard overlap between metadata-only queries and target documents was 0.010 (keyword) and 0.022 (natural language), compared with 0.027 and 0.049 for known-item queries—a 55%-63% reduction in token overlap on the synthetic corpus. This does not eliminate information leakage through the extraction step, but it substantially reduces the surface-form overlap that BM25 and lexically dependent models exploit.

Model rankings were highly stable between known-item and metadata-only queries. Kendall τ ranged from 0.59 to 0.90 across all corpus-query format combinations (mean τ=0.76), and all correlations were significant (*P=*.004; for 5 of 6 conditions *P*<.001). Spearman ρ ranged from 0.80 to 0.96 (Table 8). Rankings were most stable on MTSamples (τ=0.87-0.90) and least stable on the synthetic corpus (τ=0.59-0.77), consistent with the main study’s finding that keyword queries are more sensitive to corpus-specific vocabulary. A post hoc audit revealed that the validation script inadvertently used different HuggingFace checkpoints for 3 models (BioBERT: biobert-base-cased-v1.2 instead of biobert-v1.1; ClinicalBERT: Bio_ClinicalBERT instead of medicalai/ClinicalBERT; and GTE-base: gte-base-en-v1.5 instead of gte-base). For BioBERT and ClinicalBERT, rank stability across different checkpoints is expected: both models consistently rank in the bottom 2 positions due to embedding-geometry collapse (mean pairwise cosine similarity >0.90). For GTE-base, which ranked fourth/fifth in both experiments, the stability is more meaningful but is based on a single-model observation without formal testing.

**Table 8:** Rank stability between known-item and metadata-only queries (Kendall τ and Spearman ρ).

Corpus	Query format	τ	ρ	*P* value (τ)	*P* value (ρ)	n models
MTSamples	Keyword	0.897	0.962	<.001	<.001	13
MTSamples	Natural language	0.865	0.957	<.001	<.001	13
PMC^a^-Patients	Keyword	0.667	0.830	<.001	<.001	13
PMC-Patients	Natural language	0.744	0.863	<.001	<.001	13
Synthetic	Keyword	0.590	0.797	.004	.001	13
Synthetic	Natural language	0.769	0.907	<.001	<.001	13

^a^PMC: PubMed Central.

Absolute performance was higher under metadata-only queries (mean MRR@10=0.701) than under known-item queries (mean MRR@10=0.458), a mean increase of +0.243. This is the opposite of the expected direction: if the known-item design primarily inflated performance through lexical overlap, metadata-only queries should have produced lower scores. The most likely explanation is that metadata-only queries are cleaner and more discriminative than the original heuristic queries, which extracted raw terms from document text, including clinically irrelevant context. However, metadata-only queries may also represent a somewhat different task—more label-like and structured—which could increase separability independently of lexical overlap. The absolute score increase should therefore not be interpreted as evidence that the original queries were deficient; the primary purpose of this validation is to assess rank stability rather than to compare scores across query derivation methods. The rank stability results (above) confirm that model rankings are stable across query derivations.

BM25’s behavior was informative. On PMC-Patients with natural-language queries, BM25 dropped only slightly (0.999→0.980), confirming that its near-perfect performance was partially, but not entirely, a known-item artifact; BM25 remains genuinely strong on structured case reports. On the synthetic corpus with keyword queries, BM25 improved dramatically (0.266→0.856, Δ=+0.591), because metadata-derived keywords are clean specialty terms that BM25 matches efficiently, whereas the original document-derived keywords contained nondiscriminative clinical boilerplate.

Bottom-tier models remained at the bottom across all corpora: ClinicalBERT, BioBERT, and E5-Mistral-7B (EOS pooling) ranked last regardless of the query derivation method, confirming that the degenerate-geometry finding is robust. Top-tier models (GTE-base, Nomic-embed-text, BGE-base-en-v1.5) consistently ranked highest. These results indicate that model rank orderings are stable when queries are regenerated with reduced lexical overlap, suggesting that the benchmark’s relative conclusions are not artifacts of the specific query generation method. This does not establish generalization to arbitrary query types or clinical workflows.

## Discussion

### Principal Findings

The central finding is that clinical context variables collectively explained as much variance in retrieval performance as embedding model choice (combined η^2^=49.0% vs 47.6%). Model choice was the single largest factor (η^2^=0.408), while 2 context variables—corpus type and query format—each explained 19%-25% of the variance. The model × query format interaction (η^2^=0.029, *P*<.001) indicates that models respond differently to query type, meaning query reformulation is not model-neutral. A secondary analysis using architectural category rather than individual model identity showed that the category explained 37.2%, with the additional 3.6% captured by individual models reflecting within-category variation in training data, objectives, and configuration. The model × corpus interaction indicates that model rankings are not portable across documentation types: a team validating solely on MTSamples would have selected Nomic-embed-text, whereas a team validating on PMC-Patients would have concluded that embeddings offer limited advantage over lexical search.

The finding that rank instability was query-type–dependent adds nuance. Under keyword queries, substantial reordering occurred (Kendall τ=0.59 for MTSamples vs PMC-Patients), whereas under natural language queries, rankings were much more stable (τ=0.82-0.87). This suggests that keyword-based retrieval is particularly sensitive to corpus-specific vocabulary, whereas natural language queries allow embedding models to exploit semantic similarity more robustly across documentation types.

These results align with findings in health services research. Just as the Dartmouth Atlas demonstrated 4- to 10-fold variation in surgical procedure rates driven by clinical decision-making culture [4,5], the study’s results show that retrieval model effectiveness varies dramatically across documentation types because of differences in vocabulary, structure, and query patterns. The heterogeneity of clinical documentation across institutions [6-8] is the information retrieval analog of unwarranted clinical variation: it cannot be assumed away by choosing a better model.

An important source of within-corpus variation not directly modeled in the ANOVA is medical specialty. The 3 corpora contain documents from diverse specialties (40 in MTSamples, 20 in synthetic, and varied in PMC-Patients), and terminology, abbreviation patterns, and document structure differ substantially across specialties. The length-tercile analysis (see the “Document Length Introduces Systematic Bias” section) captures 1 dimension of within-corpus variation, but specialty-specific vocabulary differences may independently affect retrieval difficulty. In the synthetic corpus, where specialty labels are available, cross-specialty vocabulary overlap was moderate (mean pairwise Jaccard 0.266), suggesting that specialty-specific terms provide a discriminative signal for retrieval. However, the ANOVA treats each corpus as a homogeneous unit, so within-corpus specialty variation is absorbed into the residual. A per-query mixed-effects analysis with specialty as a random or fixed effect would more precisely decompose the contributions of corpus genre versus specialty, and is a priority for future work with institutional EHR data, where specialty labels are routinely available.

### Comparison With Prior Work

The finding that corpus choice significantly affects retrieval performance aligns with MIRAGE [9], which observed that no single RAG configuration dominated across medical QA datasets. This work extends MIRAGE in 3 ways: isolating the retrieval component from downstream generation, evaluating clinical documentation rather than medical examination questions, and providing a quantitative variance decomposition. The 2025 systematic review of RAG in health care [10] identified “limited evaluation frameworks” as a persistent challenge; the factorial approach directly addresses this. The finding that domain-specific encoders (BioBERT and ClinicalBERT) underperform general-purpose embeddings has precedent: contrastive training has been shown to be more important than domain-specific pretraining for retrieval tasks [16,17], and the anisotropy analysis provides a mechanistic explanation grounded in embedding geometry.

Absolute MRR@10 was higher under metadata-only queries than under the known-item design (mean Δ +0.243), with keyword queries showing a larger increase (+0.350) than natural language (+0.136). The increase was broadly distributed across models and corpora (synthetic: +0.349, PMC-Patients: +0.248, MTSamples: +0.132), indicating that heuristic keyword extraction in the main experiment produced suboptimal queries, particularly for the synthetic corpus, where specialty labels and section headers dominated the extracted terms. While absolute performance was sensitive to query formulation, relative model rankings remained stable (Kendall τ range 0.59-0.90; MTSamples keyword, *P*<.001; MTSamples natural language, *P*<.001; PMC-Patients keyword, *P*<.001; PMC-Patients natural language, *P*<.001; synthetic keyword, *P*=.004; and synthetic natural language, *P*<.001), suggesting that the main comparative conclusions are robust.

### Practical Implications for Clinical RAG

#### Practical Implications for Clinical Retrieval Model Selection and Validation

These findings yield 4 practical implications, pending validation with institutional EHR data. First, general-domain embedding leaderboards (MTEB) are insufficient for clinical model selection; E5-Mistral-7B achieves strong MTEB scores but was the worst performer here under vendor-recommended settings. Second, BM25 should always be included as a baseline and may be optimal for structured clinical documentation with high keyword density; validation with reduced lexical-dependence queries confirmed that BM25 remains genuinely strong on structured case reports (see the “Validation With Reduced-Lexical-Dependence Queries” section). Third, query format design is at least as important as model selection; organizations should invest in query reformulation before investing in expensive embedding models. Fourth, local validation against institution-specific documentation is not optional but a methodological requirement, given the model × corpus interaction (η^2^=0.040).

#### Implementation Guidance

When deploying clinical RAG, the following workflow is recommended: (1) always benchmark BM25 alongside candidate embedding models on a representative sample of local documentation; (2) test both keyword and natural-language query formats, as the optimal model may differ; (3) monitor embedding anisotropy as a diagnostic—in the data, models with anisotropy exceeding 0.85 consistently failed to provide useful retrieval, consistent with the degenerate representation problem described by Ethayarajh [26]; and (4) consider hybrid retrieval (BM25 + dense) for systems serving diverse documentation types. For initial deployment, general-purpose embeddings (BGE, GTE, and Nomic) consistently outperformed domain-specific models and are a reasonable default.

### Why BM25 Dominates Structured Case Reports

BM25’s exceptional performance on PMC-Patients (0.881 for keyword and 0.999 for natural language) reflects both the high keyword density and standardized terminology of academic case reports and, critically, the known-item retrieval design, in which queries derive from target documents. The metadata-only validation (see the “Validation With Reduced-Lexical-Dependence Queries” section) provides a nuanced picture: BM25 on PMC-Patients with natural-language queries dropped only slightly (0.999→0.980), confirming that its dominance on structured case reports is largely genuine. However, BM25 on synthetic with keyword queries increased from 0.266 to 0.856 under metadata-only queries, indicating that the original document-derived keywords contained nondiscriminative clinical boilerplate that harmed BM25 performance. The practical implication is that hybrid retrieval (BM25 + dense) may be optimal for clinical systems serving diverse documentation types, and that query quality matters as much as model choice.

### The Failure of Domain-Specific Encoders

BioBERT and ClinicalBERT’s poor performance, despite biomedical pretraining, reflects their training objective (masked language modeling) rather than the data domain. Continued pretraining with masked language modeling produced embedding spaces with anisotropy >0.90 (mean pairwise cosine similarity), making cosine similarity nondiscriminative. BioLORD-2023, trained contrastively on similar biomedical text, achieved anisotropy of 0.25-0.40 and 3-5× better performance. The training objective, not the domain, determines suitability for retrieval. This finding has practical importance: organizations should not assume that “clinical” or “biomedical” in a model name indicates suitability for clinical retrieval.

### Limitations

Several limitations should be acknowledged. First, the primary evaluation used a known-item retrieval design, in which queries are derived from target documents, increasing lexical overlap between queries and targets. Natural-language queries in the main benchmark are verbatim opening sentences from documents, producing particularly high overlap. A validation experiment using GPT-4o–generated metadata-only queries (see the “Validation With Reduced-Lexical-Dependence Queries” section) confirmed that model rankings were stable across query derivations (τ=0.59-0.90). Absolute MRR was higher under metadata-only queries, suggesting that the heuristic known-item queries did not inflate embedding model performance, although the metadata-only queries may represent a somewhat different task. External validation with authentic clinician-generated queries remains desirable.

Second, the corpora are limited to 500 documents each, which is smaller than production systems. While sufficient for detecting the large effect sizes observed, smaller effects may be missed. Third, the study evaluates single-document relevance, whereas production systems may return multiple relevant documents per query. In multidocument retrieval, chunking strategies may be more consequential than observed here: when a query requires synthesizing information across several notes (eg, longitudinal medication reconciliation or preoperative risk assessment), the ability to retrieve relevant passages from multiple documents—rather than a single matching document—may favor chunked representations that isolate topically coherent segments. The small chunking effect (η^2^=0.002) observed under the single-document paradigm may therefore understate chunking’s importance in production clinical RAG. Fourth, none of the corpora constitute actual institutional EHR data: MTSamples is a public transcription resource, PMC-Patients consists of academic case reports, and synthetic is Mistral-7B output. The synthetic corpus in particular may not fully represent real EHR data, which include templates, copy-paste errors, incomplete sentences, nonstandard abbreviations, and multiprovider documentation. Real EHR notes exhibit substantially greater structural heterogeneity than the Mistral-7B–generated notes, which follow consistent section headers and grammatically complete sentences (as reflected in the identical section-based and 512-token chunk counts in Table 2). This regularity likely understates the model × corpus interaction observed in a true production environment, where embedding models must contend with noisy, inconsistent formatting that varies both within and across institutions. The ecological validity gap is partially mitigated by the diversity across the 3 corpus types, which demonstrates that context sensitivity is present even across relatively clean public corpora—suggesting it would be amplified, not diminished, with real clinical data. Fifth, although the objective references document length and medical specialty as contextual variables, the ANOVA uses corpus type as a proxy rather than modeling these factors individually; within-corpus variation in specialty and length may account for some unexplained variance.

Sixth, the study did not include recent embedding models released in 2024-2025 (eg, GTE-Qwen2, NV-Embed, Jina-v3), nor did it evaluate cross-encoder re-ranking or hybrid retrieval strategies (BM25 + dense fusion). Of particular interest are late-chunking architectures that compute token-level embeddings over the full document context before pooling at the chunk level, potentially addressing the context fragmentation inherent in the preencoding chunking strategies evaluated here. Seventh, downstream generation quality was not evaluated, and the relationship between retrieval metrics and clinical utility remains to be established. Finally, the condition-level ANOVA treats each experimental condition as an independent observation and does not model within-condition variability. A per-query mixed-effects analysis (reported in the “Results” section) confirmed the robustness of the factor ordering, with an intraclass coefficient of 0.210 indicating that approximately one-fifth of the residual variance was attributable to between-query difficulty.

Importantly, the 3 corpora represent documentation genres (medical transcriptions, physician-authored case reports, and LLM-generated notes) rather than institutional variation. The corpus effect in the ANOVA (η^2^=0.246), therefore, reflects genre-level differences in document length, structure, and vocabulary, not cross-institutional variation (eg, differences in EHR templates, documentation norms, and specialty distributions) that would be encountered in a multisite deployment. Genre-level variation is a necessary but not sufficient condition for deployment-level generalizability, and the corpus × model interaction (η^2^=0.040) may be larger in practice when institutional variation is also present.

### Future Work

Future studies should evaluate these models on real institutional EHR data with authentic clinician-generated queries, where the structural heterogeneity of real-world documentation (copy-paste artifacts, incomplete sentences, and nonstandard abbreviations) may amplify the context-sensitivity effects observed here. Multisite validation studies—analogous to the Dartmouth Atlas approach—would test whether embedding model effectiveness varies across institutions. Hybrid retrieval strategies and cross-encoder reranking deserve systematic evaluation. Multidocument retrieval paradigms, in which queries require synthesizing information across several notes, should be investigated, as they may alter the performance hierarchy for both models and chunking strategies. Late-chunking architectures, which compute token embeddings over the full document context before pooling at the chunk level, represent a promising approach to the context-sensitivity problem identified here and warrant direct comparison. The relationship between retrieval quality and downstream clinical RAG utility should be quantified. Finally, the query-format dependence of rank stability suggests that query reformulation strategies may be a high-value intervention worthy of dedicated study.

### Conclusion

This paper presents a multicorpus benchmark of embedding models for clinical document retrieval, evaluating 10 models (plus 2 ablations and BM25) across 294 experimental conditions. A factorial ANOVA showed that clinical context variables explained as much variance in retrieval performance as embedding model choice (combined η^2^=49.0% vs 47.6%), with notable model × corpus and model × query format interactions, indicating that rankings are not portable across documentation types or query strategies. No single model dominated: Nomic-embed-text led on transcribed clinical notes, BM25 dominated on structured case reports, and retrieval was substantially harder on synthetic cross-specialty queries. Query format alone shifted MRR@10 by up to +0.659. A validation experiment using reduced-lexical-dependence queries confirmed that model rankings were stable across query derivations (τ=0.59-0.90) and that the known-item retrieval design did not inflate embedding model performance. These findings argue that the clinical informatics community should adopt local, context-specific validation as a standard requirement for RAG deployment.

## Data Availability

Code, query sets, synthetic clinical notes, full results for all 294 conditions, metadata-only validation results for 78 conditions, model configuration files, and prompt templates are available in a blinded repository for peer review (link provided in the submission system [28]). Materials will be publicly available with a persistent DOI upon acceptance. MTSamples is available online [24]. The PMC-Patients dataset is described in [25].

## Appendix

Multimedia Appendix 1 Prompt templates for Mistral-7B synthetic note generation and GPT-4o metadata extraction and query generation (validation experiment).

Multimedia Appendix 2 Model usage details, including query/document prefixes, pooling method, L2 normalization, maximum sequence length, truncation side, and library versions for all 13 retrieval configurations.

Multimedia Appendix 3 Full results table for all 294 experimental conditions.

## Abbreviations

API: application programming interface
BAAI: Beijing Academy of Artificial Intelligence
BERT: Bidirectional Encoder Representations from Transformers
BGE: BAAI General Embedding
BioBERT: Biomedical Bidirectional Encoder Representations from Transformers
BioLORD: Biomedical Learning of Ontological Representations from Definitions
BM25: Best Match 25
EHR: electronic health record
EOS: end of sequence
GPU: graphical processing unit
GTE: General Text Embeddings
LLM: large language model
MedCPT: Medical Contrastive Pre-trained Transformer
MIMIC: Medical Information Mart for Intensive Care
MIRAGE: Medical Information Retrieval-Augmented Generation Evaluation
MRR: mean reciprocal rank
MRR@10: mean reciprocal rank at 10
MTEB: Massive Text Embedding Benchmark
NDCG: normalized discounted cumulative gain
NDCG@10: normalized discounted cumulative gain at 10
P@1: precision at 1
PMC: PubMed Central
QA: question and answer
RAG: retrieval-augmented generation
