# Supplementary material for: Clinical Context Variables Collectively Rival Model Choice in Embedding-Based Retrieval: Multi-Corpus Benchmark Study
Source: JMIR Med Inform. 2026 May 7;14:e94241. doi: 10.2196/94241 (PMC13195371; doi:10.2196/94241)
Supplement: Multimedia Appendix 1 [file medinform_v14i1e94241_app1.pdf]

Multimedia Appendix 1

Prompt Templates for Synthetic Note Generation and Validation Experiment

This appendix provides the exact prompt templates used in the study. Curly-brace placeholders (eg, {specialty}) denote variable fields populated at runtime. All prompts are reproduced verbatim from the analysis scripts available in the replication repository.

1. Synthetic Clinical Note Generation (Mistral-7B-Instruct-v0.2)

Used to generate 500 synthetic clinical notes. The {specialty} placeholder was populated from a list of 20 clinical specialties, stratified to ensure uniform representation (25 notes per specialty). Temperature: 0.8. top_p: 0.9. Seed: 42.

Prompt template:

```
<s>[INST] You are a physician writing a clinical note. Write a realistic
clinical note for a {specialty} patient encounter. Include:
- Chief Complaint
- History of Present Illness (2-3 paragraphs)
- Past Medical History
- Medications
- Physical Examination findings
- Assessment and Plan

The note should be 200-400 words, use standard medical terminology and
abbreviations, and read like a real clinical document. Do not include any
patient name or identifiers. Write only the note, no commentary. [/INST]
```

2. Metadata Extraction (GPT-4o, Validation Experiment)

Used to extract structured metadata from each document in the validation experiment. The extracted fields served as input for generating reduced-lexical-dependence queries. Temperature: 0.0. Max tokens: 300. Document text was truncated to the first 3,000 characters.

Prompt template:

```
Read this clinical document and extract ONLY the following metadata fields.
Return a JSON object with exactly these keys:
{
  "specialty": "<medical specialty, e.g. Cardiology, Orthopedics>",
  "note_type": "<document type, e.g. Operative Report, Consultation Note>",
  "primary_diagnosis": "<main diagnosis or chief complaint, 2-5 words>",
  "secondary_diagnoses": ["<up to 2 additional diagnoses, 2-5 words each>"],
  "patient_demographics": "<age range and sex, e.g. elderly male>"
}
Return ONLY the JSON. No other text.

Document:
{document_text}
```

3. Natural Language Query Generation (GPT-4o, Validation Experiment)

Used to generate natural language queries from extracted metadata, ensuring queries were not derived from document text. Temperature: 0.3. Max tokens: 150.

Prompt template:

```
You are a clinician searching for a patient's clinical document.
Based ONLY on the following metadata about the document, write a natural
language clinical question that would help find this document in a search
system.

Metadata:
- Specialty: {specialty}
- Note type: {note_type}
- Primary diagnosis: {primary_diagnosis}
- Other conditions: {secondary_diagnoses}
- Patient: {patient_demographics}

Requirements:
1. Write a natural, specific clinical question (1-2 sentences)
2. Use ONLY information from the metadata above
3. Use your medical knowledge to formulate a realistic clinical question
   (you may use synonyms or related clinical terms)
4. Do NOT invent specific lab values, medications, or procedures not
   implied by the metadata

Query:
```

4. Keyword Query Generation (GPT-4o, Validation Experiment)

Used to generate keyword queries from extracted metadata. Temperature: 0.3. Max tokens: 150.

Prompt template:

```
Based ONLY on the following metadata about a clinical document,
generate 3-6 search keywords that a clinician would use to find this
document.

Metadata:
- Specialty: {specialty}
- Note type: {note_type}
- Primary diagnosis: {primary_diagnosis}
- Other conditions: {secondary_diagnoses}
- Patient: {patient_demographics}

Requirements:
1. Output only keywords separated by spaces
2. Use ONLY information derivable from the metadata
3. You may use standard medical synonyms
4. 3-6 terms total, no numbering

Keywords:
```
