# Supplementary material for: Clinical Context Variables Collectively Rival Model Choice in Embedding-Based Retrieval: Multi-Corpus Benchmark Study
Source: JMIR Med Inform. 2026 May 7;14:e94241. doi: 10.2196/94241 (PMC13195371; doi:10.2196/94241)
Supplement: Multimedia Appendix 2 [file medinform_v14i1e94241_app2.pdf]

Multimedia Appendix 2

Model Usage Details for All Retrieval Configurations

This appendix details the configuration parameters for all 13 retrieval configurations evaluated in the study (10 primary models, 2 ablation variants, and BM25). All dense models applied L2 normalization to embeddings. Library: Hugging Face Transformers and Sentence-Transformers unless otherwise noted. For BM25, the rank_bm25 Python library was used with default Okapi BM25 parameters (k1=1.5, b=0.75).

Table A1. Configuration parameters for all retrieval models.

Model	Hugging Face ID / Identifier	Category	Pooling	Query Prefix / Instruction	Document Prefix	Max Length	Dtype	Library
BioBERT	dmis-lab/biobert-v1.1	Domain Encoder	Mean	None	None	512	fp32	Transformers
ClinicalBERT	medicalai/ClinicalBERT	Domain Encoder	Mean	None	None	512	fp32	Transformers
BioLORD-2023	FremyCompany/BioLORD-2023	Biomedical Retriever	Mean	None	None	512	fp32	Transformers
MedCPT	ncbi/MedCPT-Query-Encoder ncbi/MedCPT-Article-Encoder	Biomedical Retriever	CLS	None	None	512	fp32	Transformers (dual encoder)
BGE-base-en-v1.5	BAAI/bge-base-en-v1.5	General Embedding	Mean	None	None	512	fp32	Transformers
GTE-base	thenlper/gte-base	General Embedding	Mean	None	None	512	fp32	Transformers
Nomic-embed-text	nomic-ai/nomic-embed-text-v1.5	General Embedding	Mean	"search_query: "	"search_document: "	512	fp32	Sentence-Transformers
OpenAI-emb3-small	text-embedding-3-small	General API	N/A (API)	None	None	8191	N/A	OpenAI API
E5-Mistral-7B	intfloat/e5-mistral-7b-instruct	General LLM	EOS	Instruct: "Given a clinical note, retrieve the most relevant clinical document."	None	4096	fp16	Transformers
Phi-3-mini	microsoft/Phi-3-mini-4k-instruct	General LLM	Mean	None	None	4096	fp16	Transformers
E5-Mistral-7B (ablation)	intfloat/e5-mistral-7b-instruct	General LLM	Mean (changed)	None (removed)	None	4096	fp16	Transformers
Nomic-embed-text (no prefix)	nomic-ai/nomic-embed-text-v1.5	General Embedding	Mean	None (removed)	None (removed)	512	fp32	Sentence-Transformers
BM25	rank_bm25 (Okapi)	Lexical Baseline	N/A	N/A	N/A	N/A	N/A	rank_bm25 (k1=1.5, b=0.75)

Yellow rows = ablation variants; green row = lexical baseline. All dense models applied L2 normalization to output embeddings. MedCPT uses separate encoder models for queries and documents (dual encoder architecture). The E5-Mistral-7B ablation changes pooling from EOS to mean and removes the instruction prefix. The Nomic ablation removes both query and document prefixes. Truncation is applied to the right (end) of the input for all models.
