# Supplementary material for: Clinical Context Variables Collectively Rival Model Choice in Embedding-Based Retrieval: Multi-Corpus Benchmark Study
Source: JMIR Med Inform. 2026 May 7;14:e94241. doi: 10.2196/94241 (PMC13195371; doi:10.2196/94241)
Supplement: Multimedia Appendix 3 [file medinform_v14i1e94241_app3.pdf]

Multimedia Appendix 3

Full Results for All 294 Experimental Conditions

This appendix presents the complete results for all 294 experimental conditions (288 balanced embedding conditions + 6 BM25 conditions). Results are also available in CSV format in the replication repository. Due to the width of the data, the table is split into identification columns and metric columns across pages.

Table A2a. Experimental conditions and primary metrics.

Model	Category	Corpus	Query Format	Chunk Strategy	MRR@10	Recall@10	Hit@10
BM25	Lexical Baseline	MTS	KW	full	0.694	0.862	0.862
BM25	Lexical Baseline	MTS	NL	full	0.883	0.938	0.938
BM25	Lexical Baseline	PMC	KW	full	0.881	0.960	0.960
BM25	Lexical Baseline	PMC	NL	full	0.999	1.000	1.000
BM25	Lexical Baseline	Synth	KW	full	0.266	0.508	0.508
BM25	Lexical Baseline	Synth	NL	full	0.630	0.842	0.842
BioBERT	Domain Encoder	MTS	KW	full	0.165	0.362	0.362
BioBERT	Domain Encoder	MTS	NL	full	0.310	0.530	0.530
BioBERT	Domain Encoder	MTS	KW	f256	0.157	0.382	0.382
BioBERT	Domain Encoder	MTS	NL	f256	0.406	0.644	0.644
BioBERT	Domain Encoder	MTS	KW	f512	0.128	0.310	0.310
BioBERT	Domain Encoder	MTS	NL	f512	0.263	0.506	0.506
BioBERT	Domain Encoder	MTS	KW	section	0.165	0.362	0.362
BioBERT	Domain Encoder	MTS	NL	section	0.310	0.530	0.530
BioBERT	Domain Encoder	PMC	KW	full	0.148	0.306	0.306
BioBERT	Domain Encoder	PMC	NL	full	0.474	0.684	0.684
BioBERT	Domain Encoder	PMC	KW	f256	0.103	0.232	0.232
BioBERT	Domain Encoder	PMC	NL	f256	0.795	0.884	0.884
BioBERT	Domain Encoder	PMC	KW	f512	0.066	0.166	0.166
BioBERT	Domain Encoder	PMC	NL	f512	0.468	0.686	0.686
BioBERT	Domain Encoder	PMC	KW	section	0.148	0.306	0.306
BioBERT	Domain Encoder	PMC	NL	section	0.474	0.684	0.684
BioBERT	Domain Encoder	Synth	KW	full	0.040	0.110	0.110
BioBERT	Domain Encoder	Synth	NL	full	0.102	0.248	0.248
BioBERT	Domain Encoder	Synth	KW	f256	0.036	0.086	0.086
BioBERT	Domain Encoder	Synth	NL	f256	0.126	0.310	0.310
BioBERT	Domain Encoder	Synth	KW	f512	0.029	0.086	0.086
BioBERT	Domain Encoder	Synth	NL	f512	0.058	0.156	0.156
BioBERT	Domain Encoder	Synth	KW	section	0.020	0.068	0.068
BioBERT	Domain Encoder	Synth	NL	section	0.071	0.154	0.154
ClinicalBERT	Domain Encoder	MTS	KW	full	0.129	0.314	0.314
ClinicalBERT	Domain Encoder	MTS	NL	full	0.278	0.536	0.536
ClinicalBERT	Domain Encoder	MTS	KW	f256	0.119	0.292	0.292
ClinicalBERT	Domain Encoder	MTS	NL	f256	0.324	0.570	0.570
ClinicalBERT	Domain Encoder	MTS	KW	f512	0.089	0.260	0.260
ClinicalBERT	Domain Encoder	MTS	NL	f512	0.215	0.452	0.452
ClinicalBERT	Domain Encoder	MTS	KW	section	0.129	0.314	0.314
ClinicalBERT	Domain Encoder	MTS	NL	section	0.278	0.536	0.536
ClinicalBERT	Domain Encoder	PMC	KW	full	0.046	0.088	0.088
ClinicalBERT	Domain Encoder	PMC	NL	full	0.376	0.584	0.584
ClinicalBERT	Domain Encoder	PMC	KW	f256	0.024	0.066	0.066

Model	Category	Corpus	Query Form at	Chunk Strategy	MRR@10	Recall@10	Hit@10
ClinicalBERT	Domain Encoder	PMC	NL	f256	0.710	0.830	0.830
ClinicalBERT	Domain Encoder	PMC	KW	f512	0.020	0.054	0.054
ClinicalBERT	Domain Encoder	PMC	NL	f512	0.370	0.588	0.588
ClinicalBERT	Domain Encoder	PMC	KW	section	0.046	0.088	0.088
ClinicalBERT	Domain Encoder	PMC	NL	section	0.376	0.584	0.584
ClinicalBERT	Domain Encoder	Synth	KW	full	0.014	0.046	0.046
ClinicalBERT	Domain Encoder	Synth	NL	full	0.098	0.230	0.230
ClinicalBERT	Domain Encoder	Synth	KW	f256	0.018	0.052	0.052
ClinicalBERT	Domain Encoder	Synth	NL	f256	0.137	0.308	0.308
ClinicalBERT	Domain Encoder	Synth	KW	f512	0.011	0.036	0.036
ClinicalBERT	Domain Encoder	Synth	NL	f512	0.058	0.152	0.152
ClinicalBERT	Domain Encoder	Synth	KW	section	0.009	0.030	0.030
ClinicalBERT	Domain Encoder	Synth	NL	section	0.076	0.174	0.174
BioLORD-2023	Biomedical Retriever	MTS	KW	full	0.581	0.830	0.830
BioLORD-2023	Biomedical Retriever	MTS	NL	full	0.768	0.924	0.924
BioLORD-2023	Biomedical Retriever	MTS	KW	f256	0.561	0.808	0.808
BioLORD-2023	Biomedical Retriever	MTS	NL	f256	0.814	0.928	0.928
BioLORD-2023	Biomedical Retriever	MTS	KW	f512	0.564	0.806	0.806
BioLORD-2023	Biomedical Retriever	MTS	NL	f512	0.775	0.922	0.922
BioLORD-2023	Biomedical Retriever	MTS	KW	section	0.581	0.830	0.830
BioLORD-2023	Biomedical Retriever	MTS	NL	section	0.768	0.924	0.924
BioLORD-2023	Biomedical Retriever	PMC	KW	full	0.225	0.416	0.416
BioLORD-2023	Biomedical Retriever	PMC	NL	full	0.884	0.964	0.964
BioLORD-2023	Biomedical Retriever	PMC	KW	f256	0.193	0.358	0.358
BioLORD-2023	Biomedical Retriever	PMC	NL	f256	0.937	0.982	0.982
BioLORD-2023	Biomedical Retriever	PMC	KW	f512	0.193	0.338	0.338
BioLORD-2023	Biomedical Retriever	PMC	NL	f512	0.882	0.962	0.962
BioLORD-2023	Biomedical Retriever	PMC	KW	section	0.225	0.416	0.416
BioLORD-2023	Biomedical Retriever	PMC	NL	section	0.884	0.964	0.964
BioLORD-2023	Biomedical Retriever	Synth	KW	full	0.162	0.456	0.456
BioLORD-2023	Biomedical Retriever	Synth	NL	full	0.419	0.686	0.686
BioLORD-2023	Biomedical Retriever	Synth	KW	f256	0.163	0.418	0.418
BioLORD-2023	Biomedical Retriever	Synth	NL	f256	0.472	0.696	0.696
BioLORD-2023	Biomedical Retriever	Synth	KW	f512	0.137	0.388	0.388

Model	Category	Corpus	Query Form at	Chunk Strategy	MRR@10	Recall@10	Hit@10
BioLORD-2023	Biomedical Retriever	Synth	NL	f512	0.398	0.668	0.668
BioLORD-2023	Biomedical Retriever	Synth	KW	section	0.133	0.398	0.398
BioLORD-2023	Biomedical Retriever	Synth	NL	section	0.407	0.660	0.660
MedCPT	Biomedical Retriever	MTS	KW	full	0.624	0.856	0.856
MedCPT	Biomedical Retriever	MTS	NL	full	0.725	0.912	0.912
MedCPT	Biomedical Retriever	MTS	KW	f256	0.642	0.868	0.868
MedCPT	Biomedical Retriever	MTS	NL	f256	0.766	0.926	0.926
MedCPT	Biomedical Retriever	MTS	KW	f512	0.630	0.848	0.848
MedCPT	Biomedical Retriever	MTS	NL	f512	0.726	0.918	0.918
MedCPT	Biomedical Retriever	MTS	KW	section	0.624	0.856	0.856
MedCPT	Biomedical Retriever	MTS	NL	section	0.725	0.912	0.912
MedCPT	Biomedical Retriever	PMC	KW	full	0.569	0.752	0.752
MedCPT	Biomedical Retriever	PMC	NL	full	0.720	0.898	0.898
MedCPT	Biomedical Retriever	PMC	KW	f256	0.568	0.766	0.766
MedCPT	Biomedical Retriever	PMC	NL	f256	0.810	0.946	0.946
MedCPT	Biomedical Retriever	PMC	KW	f512	0.536	0.728	0.728
MedCPT	Biomedical Retriever	PMC	NL	f512	0.713	0.892	0.892
MedCPT	Biomedical Retriever	PMC	KW	section	0.569	0.752	0.752
MedCPT	Biomedical Retriever	PMC	NL	section	0.720	0.898	0.898
MedCPT	Biomedical Retriever	Synth	KW	full	0.212	0.550	0.550
MedCPT	Biomedical Retriever	Synth	NL	full	0.201	0.460	0.460
MedCPT	Biomedical Retriever	Synth	KW	f256	0.196	0.514	0.514
MedCPT	Biomedical Retriever	Synth	NL	f256	0.189	0.432	0.432
MedCPT	Biomedical Retriever	Synth	KW	f512	0.210	0.548	0.548
MedCPT	Biomedical Retriever	Synth	NL	f512	0.200	0.454	0.454
MedCPT	Biomedical Retriever	Synth	KW	section	0.214	0.552	0.552
MedCPT	Biomedical Retriever	Synth	NL	section	0.202	0.458	0.458
BGE-base	General Embedding	MTS	KW	full	0.759	0.906	0.906
BGE-base	General Embedding	MTS	NL	full	0.882	0.964	0.964

Model	Category	Corpus	Query Format	Chunk Strategy	MRR@10	Recall@10	Hit@10
BGE-base	General Embedding	MTS	KW	f256	0.735	0.900	0.900
BGE-base	General Embedding	MTS	NL	f256	0.888	0.968	0.968
BGE-base	General Embedding	MTS	KW	f512	0.760	0.906	0.906
BGE-base	General Embedding	MTS	NL	f512	0.888	0.962	0.962
BGE-base	General Embedding	MTS	KW	section	0.759	0.906	0.906
BGE-base	General Embedding	MTS	NL	section	0.882	0.964	0.964
BGE-base	General Embedding	PMC	KW	full	0.459	0.658	0.658
BGE-base	General Embedding	PMC	NL	full	0.883	0.962	0.962
BGE-base	General Embedding	PMC	KW	f256	0.441	0.610	0.610
BGE-base	General Embedding	PMC	NL	f256	0.896	0.966	0.966
BGE-base	General Embedding	PMC	KW	f512	0.379	0.550	0.550
BGE-base	General Embedding	PMC	NL	f512	0.877	0.954	0.954
BGE-base	General Embedding	PMC	KW	section	0.459	0.658	0.658
BGE-base	General Embedding	PMC	NL	section	0.883	0.962	0.962
BGE-base	General Embedding	Synth	KW	full	0.253	0.582	0.582
BGE-base	General Embedding	Synth	NL	full	0.494	0.728	0.728
BGE-base	General Embedding	Synth	KW	f256	0.245	0.582	0.582
BGE-base	General Embedding	Synth	NL	f256	0.479	0.708	0.708
BGE-base	General Embedding	Synth	KW	f512	0.236	0.566	0.566
BGE-base	General Embedding	Synth	NL	f512	0.486	0.716	0.716
BGE-base	General Embedding	Synth	KW	section	0.238	0.572	0.572
BGE-base	General Embedding	Synth	NL	section	0.477	0.706	0.706
GTE-base	General Embedding	MTS	KW	full	0.730	0.904	0.904
GTE-base	General Embedding	MTS	NL	full	0.885	0.966	0.966
GTE-base	General Embedding	MTS	KW	f256	0.724	0.888	0.888
GTE-base	General Embedding	MTS	NL	f256	0.901	0.970	0.970
GTE-base	General Embedding	MTS	KW	f512	0.721	0.914	0.914
GTE-base	General Embedding	MTS	NL	f512	0.892	0.970	0.970
GTE-base	General Embedding	MTS	KW	section	0.730	0.904	0.904

Model	Category	Corpus	Query Form at	Chunk Strategy	MRR@10	Recall@10	Hit@10
GTE-base	General Embedding	MTS	NL	section	0.885	0.966	0.966
GTE-base	General Embedding	PMC	KW	full	0.413	0.614	0.614
GTE-base	General Embedding	PMC	NL	full	0.902	0.978	0.978
GTE-base	General Embedding	PMC	KW	f256	0.358	0.522	0.522
GTE-base	General Embedding	PMC	NL	f256	0.897	0.966	0.966
GTE-base	General Embedding	PMC	KW	f512	0.307	0.476	0.476
GTE-base	General Embedding	PMC	NL	f512	0.889	0.970	0.970
GTE-base	General Embedding	PMC	KW	section	0.413	0.614	0.614
GTE-base	General Embedding	PMC	NL	section	0.902	0.978	0.978
GTE-base	General Embedding	Synth	KW	full	0.219	0.518	0.518
GTE-base	General Embedding	Synth	NL	full	0.469	0.698	0.698
GTE-base	General Embedding	Synth	KW	f256	0.198	0.504	0.504
GTE-base	General Embedding	Synth	NL	f256	0.451	0.674	0.674
GTE-base	General Embedding	Synth	KW	f512	0.192	0.468	0.468
GTE-base	General Embedding	Synth	NL	f512	0.447	0.668	0.668
GTE-base	General Embedding	Synth	KW	section	0.199	0.488	0.488
GTE-base	General Embedding	Synth	NL	section	0.448	0.666	0.666
Nomic-embed-text	General Embedding	MTS	KW	full	0.768	0.894	0.894
Nomic-embed-text	General Embedding	MTS	NL	full	0.889	0.960	0.960
Nomic-embed-text	General Embedding	MTS	KW	f256	0.745	0.882	0.882
Nomic-embed-text	General Embedding	MTS	NL	f256	0.891	0.952	0.952
Nomic-embed-text	General Embedding	MTS	KW	f512	0.754	0.880	0.880
Nomic-embed-text	General Embedding	MTS	NL	f512	0.889	0.956	0.956
Nomic-embed-text	General Embedding	MTS	KW	section	0.768	0.894	0.894
Nomic-embed-text	General Embedding	MTS	NL	section	0.889	0.960	0.960
Nomic-embed-text	General Embedding	PMC	KW	full	0.460	0.632	0.632
Nomic-embed-text	General Embedding	PMC	NL	full	0.950	0.986	0.986
Nomic-embed-text	General Embedding	PMC	KW	f256	0.460	0.626	0.626
Nomic-embed-text	General Embedding	PMC	NL	f256	0.967	0.990	0.990

Model	Category	Corpus	Query Form at	Chunk Strategy	MRR@10	Recall@10	Hit@10
Nomic-embed-text	General Embedding	PMC	KW	f512	0.406	0.574	0.574
Nomic-embed-text	General Embedding	PMC	NL	f512	0.943	0.988	0.988
Nomic-embed-text	General Embedding	PMC	KW	section	0.461	0.632	0.632
Nomic-embed-text	General Embedding	PMC	NL	section	0.950	0.986	0.986
Nomic-embed-text	General Embedding	Synth	KW	full	0.288	0.620	0.620
Nomic-embed-text	General Embedding	Synth	NL	full	0.510	0.724	0.724
Nomic-embed-text	General Embedding	Synth	KW	f256	0.258	0.582	0.582
Nomic-embed-text	General Embedding	Synth	NL	f256	0.498	0.710	0.710
Nomic-embed-text	General Embedding	Synth	KW	f512	0.283	0.606	0.606
Nomic-embed-text	General Embedding	Synth	NL	f512	0.507	0.718	0.718
Nomic-embed-text	General Embedding	Synth	KW	section	0.270	0.588	0.588
Nomic-embed-text	General Embedding	Synth	NL	section	0.496	0.718	0.718
OpenAI-emb3-smal l	General API	MTS	KW	full	0.711	0.918	0.918
OpenAI-emb3-smal l	General API	MTS	NL	full	0.862	0.968	0.968
OpenAI-emb3-smal l	General API	MTS	KW	f256	0.742	0.916	0.916
OpenAI-emb3-smal l	General API	MTS	NL	f256	0.890	0.964	0.964
OpenAI-emb3-smal l	General API	MTS	KW	f512	0.723	0.912	0.912
OpenAI-emb3-smal l	General API	MTS	NL	f512	0.874	0.962	0.962
OpenAI-emb3-smal l	General API	MTS	KW	section	0.710	0.918	0.918
OpenAI-emb3-smal l	General API	MTS	NL	section	0.860	0.968	0.968
OpenAI-emb3-smal l	General API	PMC	KW	full	0.410	0.612	0.612
OpenAI-emb3-smal l	General API	PMC	NL	full	0.829	0.932	0.932
OpenAI-emb3-smal l	General API	PMC	KW	f256	0.481	0.664	0.664
OpenAI-emb3-smal l	General API	PMC	NL	f256	0.987	0.996	0.996
OpenAI-emb3-smal l	General API	PMC	KW	f512	0.433	0.636	0.636
OpenAI-emb3-smal l	General API	PMC	NL	f512	0.942	0.988	0.988
OpenAI-emb3-smal l	General API	PMC	KW	section	0.409	0.614	0.614
OpenAI-emb3-smal l	General API	PMC	NL	section	0.828	0.932	0.932
OpenAI-emb3-smal l	General API	Synth	KW	full	0.273	0.650	0.650

Model	Category	Corpus	Query Format	Chunk Strategy	MRR@10	Recall@10	Hit@10
OpenAI-emb3-small	General API	Synth	NL	full	0.454	0.682	0.682
OpenAI-emb3-small	General API	Synth	KW	f256	0.272	0.636	0.636
OpenAI-emb3-small	General API	Synth	NL	f256	0.495	0.714	0.714
OpenAI-emb3-small	General API	Synth	KW	f512	0.244	0.606	0.606
OpenAI-emb3-small	General API	Synth	NL	f512	0.412	0.678	0.678
OpenAI-emb3-small	General API	Synth	KW	section	0.282	0.652	0.652
OpenAI-emb3-small	General API	Synth	NL	section	0.454	0.678	0.678
E5-Mistral-7B	General LLM	MTS	KW	full	0.062	0.142	0.142
E5-Mistral-7B	General LLM	MTS	NL	full	0.158	0.302	0.302
E5-Mistral-7B	General LLM	MTS	KW	f256	0.139	0.286	0.286
E5-Mistral-7B	General LLM	MTS	NL	f256	0.252	0.434	0.434
E5-Mistral-7B	General LLM	MTS	KW	f512	0.089	0.196	0.196
E5-Mistral-7B	General LLM	MTS	NL	f512	0.228	0.388	0.388
E5-Mistral-7B	General LLM	MTS	KW	section	0.062	0.142	0.142
E5-Mistral-7B	General LLM	MTS	NL	section	0.158	0.302	0.302
E5-Mistral-7B	General LLM	PMC	KW	full	0.169	0.314	0.314
E5-Mistral-7B	General LLM	PMC	NL	full	0.226	0.500	0.500
E5-Mistral-7B	General LLM	PMC	KW	f256	0.120	0.256	0.256
E5-Mistral-7B	General LLM	PMC	NL	f256	0.054	0.120	0.120
E5-Mistral-7B	General LLM	PMC	KW	f512	0.121	0.242	0.242
E5-Mistral-7B	General LLM	PMC	NL	f512	0.064	0.140	0.140
E5-Mistral-7B	General LLM	PMC	KW	section	0.169	0.314	0.314
E5-Mistral-7B	General LLM	PMC	NL	section	0.226	0.500	0.500
E5-Mistral-7B	General LLM	Synth	KW	full	0.042	0.120	0.120
E5-Mistral-7B	General LLM	Synth	NL	full	0.041	0.100	0.100
E5-Mistral-7B	General LLM	Synth	KW	f256	0.027	0.080	0.080
E5-Mistral-7B	General LLM	Synth	NL	f256	0.043	0.094	0.094
E5-Mistral-7B	General LLM	Synth	KW	f512	0.040	0.096	0.096
E5-Mistral-7B	General LLM	Synth	NL	f512	0.041	0.106	0.106
E5-Mistral-7B	General LLM	Synth	KW	section	0.049	0.144	0.144
E5-Mistral-7B	General LLM	Synth	NL	section	0.073	0.218	0.218
Phi-3-mini	General LLM	MTS	KW	full	0.175	0.394	0.394
Phi-3-mini	General LLM	MTS	NL	full	0.532	0.786	0.786
Phi-3-mini	General LLM	MTS	KW	f256	0.189	0.398	0.398
Phi-3-mini	General LLM	MTS	NL	f256	0.538	0.754	0.754
Phi-3-mini	General LLM	MTS	KW	f512	0.189	0.392	0.392
Phi-3-mini	General LLM	MTS	NL	f512	0.549	0.756	0.756
Phi-3-mini	General LLM	MTS	KW	section	0.175	0.394	0.394
Phi-3-mini	General LLM	MTS	NL	section	0.532	0.786	0.786
Phi-3-mini	General LLM	PMC	KW	full	0.140	0.284	0.284
Phi-3-mini	General LLM	PMC	NL	full	0.578	0.754	0.754
Phi-3-mini	General LLM	PMC	KW	f256	0.108	0.222	0.222
Phi-3-mini	General LLM	PMC	NL	f256	0.834	0.910	0.910
Phi-3-mini	General LLM	PMC	KW	f512	0.097	0.218	0.218
Phi-3-mini	General LLM	PMC	NL	f512	0.668	0.828	0.828

Model	Category	Corpus	Query Form at	Chunk Strategy	MRR@10	Recall@10	Hit@10
Phi-3-mini	General LLM	PMC	KW	section	0.140	0.286	0.286
Phi-3-mini	General LLM	PMC	NL	section	0.579	0.756	0.756
Phi-3-mini	General LLM	Synth	KW	full	0.031	0.092	0.092
Phi-3-mini	General LLM	Synth	NL	full	0.123	0.288	0.288
Phi-3-mini	General LLM	Synth	KW	f256	0.031	0.080	0.080
Phi-3-mini	General LLM	Synth	NL	f256	0.145	0.318	0.318
Phi-3-mini	General LLM	Synth	KW	f512	0.030	0.072	0.072
Phi-3-mini	General LLM	Synth	NL	f512	0.114	0.268	0.268
Phi-3-mini	General LLM	Synth	KW	section	0.028	0.088	0.088
Phi-3-mini	General LLM	Synth	NL	section	0.114	0.274	0.274
E5-Mistral-7B-ablation	General LLM	MTS	KW	full	0.344	0.670	0.670
E5-Mistral-7B-ablation	General LLM	MTS	NL	full	0.677	0.872	0.872
E5-Mistral-7B-ablation	General LLM	MTS	KW	f256	0.278	0.552	0.552
E5-Mistral-7B-ablation	General LLM	MTS	NL	f256	0.630	0.844	0.844
E5-Mistral-7B-ablation	General LLM	MTS	KW	f512	0.273	0.580	0.580
E5-Mistral-7B-ablation	General LLM	MTS	NL	f512	0.643	0.826	0.826
E5-Mistral-7B-ablation	General LLM	MTS	KW	section	0.344	0.670	0.670
E5-Mistral-7B-ablation	General LLM	MTS	NL	section	0.677	0.872	0.872
E5-Mistral-7B-ablation	General LLM	PMC	KW	full	0.195	0.340	0.340
E5-Mistral-7B-ablation	General LLM	PMC	NL	full	0.638	0.786	0.786
E5-Mistral-7B-ablation	General LLM	PMC	KW	f256	0.127	0.262	0.262
E5-Mistral-7B-ablation	General LLM	PMC	NL	f256	0.881	0.942	0.942
E5-Mistral-7B-ablation	General LLM	PMC	KW	f512	0.089	0.204	0.204
E5-Mistral-7B-ablation	General LLM	PMC	NL	f512	0.742	0.864	0.864
E5-Mistral-7B-ablation	General LLM	PMC	KW	section	0.195	0.340	0.340
E5-Mistral-7B-ablation	General LLM	PMC	NL	section	0.638	0.788	0.788
E5-Mistral-7B-ablation	General LLM	Synth	KW	full	0.152	0.432	0.432
E5-Mistral-7B-ablation	General LLM	Synth	NL	full	0.305	0.560	0.560
E5-Mistral-7B-ablation	General LLM	Synth	KW	f256	0.105	0.306	0.306
E5-Mistral-7B-ablation	General LLM	Synth	NL	f256	0.320	0.580	0.580
E5-Mistral-7B-ablation	General LLM	Synth	KW	f512	0.124	0.380	0.380
E5-Mistral-7B-ablation	General LLM	Synth	NL	f512	0.265	0.532	0.532
E5-Mistral-7B-ablation	General LLM	Synth	KW	section	0.107	0.340	0.340

Model	Category	Corpus	Query Format	Chunk Strategy	MRR@10	Recall@10	Hit@10
E5-Mistral-7B-ablation	General LLM	Synth	NL	section	0.251	0.524	0.524
Nomic-embed-text-nopfx	General Embedding	MTS	KW	full	0.746	0.888	0.888
Nomic-embed-text-nopfx	General Embedding	MTS	NL	full	0.884	0.954	0.954
Nomic-embed-text-nopfx	General Embedding	MTS	KW	f256	0.709	0.854	0.854
Nomic-embed-text-nopfx	General Embedding	MTS	NL	f256	0.890	0.950	0.950
Nomic-embed-text-nopfx	General Embedding	MTS	KW	f512	0.724	0.878	0.878
Nomic-embed-text-nopfx	General Embedding	MTS	NL	f512	0.895	0.952	0.952
Nomic-embed-text-nopfx	General Embedding	MTS	KW	section	0.746	0.888	0.888
Nomic-embed-text-nopfx	General Embedding	MTS	NL	section	0.884	0.954	0.954
Nomic-embed-text-nopfx	General Embedding	PMC	KW	full	0.399	0.598	0.598
Nomic-embed-text-nopfx	General Embedding	PMC	NL	full	0.959	0.990	0.990
Nomic-embed-text-nopfx	General Embedding	PMC	KW	f256	0.318	0.526	0.526
Nomic-embed-text-nopfx	General Embedding	PMC	NL	f256	0.961	0.990	0.990
Nomic-embed-text-nopfx	General Embedding	PMC	KW	f512	0.293	0.476	0.476
Nomic-embed-text-nopfx	General Embedding	PMC	NL	f512	0.954	0.990	0.990
Nomic-embed-text-nopfx	General Embedding	PMC	KW	section	0.399	0.598	0.598
Nomic-embed-text-nopfx	General Embedding	PMC	NL	section	0.959	0.990	0.990
Nomic-embed-text-nopfx	General Embedding	Synth	KW	full	0.314	0.658	0.658
Nomic-embed-text-nopfx	General Embedding	Synth	NL	full	0.503	0.718	0.718
Nomic-embed-text-nopfx	General Embedding	Synth	KW	f256	0.291	0.634	0.634
Nomic-embed-text-nopfx	General Embedding	Synth	NL	f256	0.512	0.710	0.710
Nomic-embed-text-nopfx	General Embedding	Synth	KW	f512	0.301	0.642	0.642
Nomic-embed-text-nopfx	General Embedding	Synth	NL	f512	0.502	0.712	0.712
Nomic-embed-text-nopfx	General Embedding	Synth	KW	section	0.298	0.636	0.636
Nomic-embed-text-nopfx	General Embedding	Synth	NL	section	0.497	0.720	0.720

Table A2b. Extended retrieval metrics.

Model	Corpus	Query	Chunk	MRR@1	MRR@5	MRR@10	MRR@20	R@1	R@5	R@10	R@20	R@50	R@100
BM25	MTS	KW	full	0.618	0.684	0.694	0.697	0.618	0.788	0.862	0.906	0.948	0.962
BM25	MTS	NL	full	0.856	0.881	0.883	0.884	0.856	0.922	0.938	0.944	0.960	0.966

Model	Corpus	Query	Chunk	MRR@1	MRR@5	MRR@10	MRR@20	R@1	R@5	R@10	R@20	R@50	R@100
BM25	PMC	KW	full	0.838	0.878	0.881	0.882	0.838	0.938	0.960	0.976	0.992	1.000
BM25	PMC	NL	full	0.998	0.999	0.999	0.999	0.998	1.000	1.000	1.000	1.000	1.000
BM25	Syn	KW	full	0.166	0.251	0.266	0.273	0.166	0.396	0.508	0.614	0.772	0.892
BM25	Syn	NL	full	0.520	0.621	0.630	0.634	0.520	0.778	0.842	0.908	0.990	1.000
BioBERT	MTS	KW	full	0.090	0.154	0.165	0.172	0.090	0.272	0.362	0.460	0.604	0.714
BioBERT	MTS	NL	full	0.218	0.297	0.310	0.317	0.218	0.432	0.530	0.624	0.754	0.814
BioBERT	MTS	KW	f256	0.082	0.140	0.157	0.166	0.082	0.256	0.382	0.500	0.656	0.780
BioBERT	MTS	NL	f256	0.310	0.390	0.406	0.412	0.310	0.524	0.644	0.728	0.820	0.894
BioBERT	MTS	KW	f512	0.066	0.114	0.128	0.136	0.066	0.210	0.310	0.434	0.590	0.726
BioBERT	MTS	NL	f512	0.170	0.245	0.263	0.270	0.170	0.374	0.506	0.612	0.734	0.810
BioBERT	MTS	KW	section	0.090	0.154	0.165	0.172	0.090	0.272	0.362	0.460	0.604	0.714
BioBERT	MTS	NL	section	0.218	0.297	0.310	0.317	0.218	0.432	0.530	0.624	0.754	0.814
BioBERT	PMC	KW	full	0.090	0.138	0.148	0.153	0.090	0.232	0.306	0.388	0.496	0.642
BioBERT	PMC	NL	full	0.388	0.461	0.474	0.480	0.388	0.588	0.684	0.768	0.852	0.922
BioBERT	PMC	KW	f256	0.058	0.094	0.103	0.110	0.058	0.162	0.232	0.324	0.466	0.594
BioBERT	PMC	NL	f256	0.744	0.792	0.795	0.797	0.744	0.862	0.884	0.914	0.948	0.976
BioBERT	PMC	KW	f512	0.036	0.058	0.066	0.072	0.036	0.100	0.166	0.250	0.424	0.566
BioBERT	PMC	NL	f512	0.382	0.455	0.468	0.474	0.382	0.584	0.686	0.770	0.850	0.926
BioBERT	PMC	KW	section	0.090	0.138	0.148	0.153	0.090	0.232	0.306	0.388	0.496	0.640
BioBERT	PMC	NL	section	0.388	0.461	0.474	0.480	0.388	0.588	0.684	0.768	0.852	0.922
BioBERT	Syn	KW	full	0.020	0.034	0.040	0.044	0.020	0.064	0.110	0.166	0.280	0.410
BioBERT	Syn	NL	full	0.056	0.090	0.102	0.111	0.056	0.154	0.248	0.366	0.538	0.718
BioBERT	Syn	KW	f256	0.022	0.032	0.036	0.039	0.022	0.052	0.086	0.134	0.234	0.384
BioBERT	Syn	NL	f256	0.068	0.113	0.126	0.132	0.068	0.208	0.310	0.396	0.562	0.750
BioBERT	Syn	KW	f512	0.012	0.024	0.029	0.034	0.012	0.050	0.086	0.152	0.266	0.396
BioBERT	Syn	NL	f512	0.032	0.049	0.058	0.063	0.032	0.088	0.156	0.226	0.400	0.598
BioBERT	Syn	KW	section	0.008	0.016	0.020	0.024	0.008	0.036	0.068	0.126	0.216	0.382
BioBERT	Syn	NL	section	0.044	0.064	0.071	0.076	0.044	0.102	0.154	0.236	0.446	0.640
ClinicalBERT	MTS	KW	full	0.066	0.116	0.129	0.139	0.066	0.216	0.314	0.442	0.614	0.734
ClinicalBERT	MTS	NL	full	0.176	0.262	0.278	0.287	0.176	0.416	0.536	0.658	0.778	0.858
ClinicalBERT	MTS	KW	f256	0.062	0.106	0.119	0.127	0.062	0.194	0.292	0.410	0.568	0.702
ClinicalBERT	MTS	NL	f256	0.224	0.309	0.324	0.332	0.224	0.458	0.570	0.674	0.800	0.866
ClinicalBERT	MTS	KW	f512	0.038	0.076	0.089	0.098	0.038	0.156	0.260	0.382	0.576	0.740
ClinicalBERT	MTS	NL	f512	0.130	0.200	0.215	0.224	0.130	0.330	0.452	0.574	0.742	0.836
ClinicalBERT	MTS	KW	section	0.066	0.116	0.129	0.139	0.066	0.216	0.314	0.442	0.614	0.734
ClinicalBERT	MTS	NL	section	0.176	0.262	0.278	0.287	0.176	0.416	0.536	0.658	0.778	0.858
ClinicalBERT	PMC	KW	full	0.030	0.043	0.046	0.051	0.030	0.068	0.088	0.158	0.304	0.454
ClinicalBERT	PMC	NL	full	0.286	0.363	0.376	0.383	0.286	0.492	0.584	0.694	0.802	0.876
ClinicalBERT	PMC	KW	f256	0.012	0.020	0.024	0.027	0.012	0.036	0.066	0.110	0.196	0.316
ClinicalBERT	PMC	NL	f256	0.658	0.704	0.710	0.713	0.658	0.784	0.830	0.878	0.942	0.956
ClinicalBERT	PMC	KW	f512	0.008	0.017	0.020	0.022	0.008	0.036	0.054	0.088	0.168	0.304
ClinicalBERT	PMC	NL	f512	0.282	0.357	0.370	0.376	0.282	0.488	0.588	0.682	0.786	0.858
ClinicalBERT	PMC	KW	section	0.030	0.043	0.046	0.051	0.030	0.068	0.088	0.158	0.302	0.454
ClinicalBERT	PMC	NL	section	0.286	0.363	0.376	0.383	0.286	0.492	0.584	0.694	0.802	0.876
ClinicalBERT	Syn	KW	full	0.006	0.011	0.014	0.016	0.006	0.024	0.046	0.076	0.166	0.314
ClinicalBERT	Syn	NL	full	0.052	0.086	0.098	0.105	0.052	0.142	0.230	0.334	0.542	0.696
ClinicalBERT	Syn	KW	f256	0.006	0.016	0.018	0.020	0.006	0.034	0.052	0.082	0.154	0.286
ClinicalBERT	Syn	NL	f256	0.082	0.123	0.137	0.143	0.082	0.202	0.308	0.404	0.580	0.722

Model	Corpus	Query	Chunk	MRR@1	MRR@5	MRR@10	MRR@20	R@1	R@5	R@10	R@20	R@50	R@100
ClinicalBERT	Syn	KW	f512	0.006	0.008	0.011	0.013	0.006	0.012	0.036	0.060	0.136	0.284
ClinicalBERT	Syn	NL	f512	0.028	0.050	0.058	0.064	0.028	0.090	0.152	0.250	0.404	0.554
ClinicalBERT	Syn	KW	section	0.002	0.007	0.009	0.011	0.002	0.014	0.030	0.060	0.140	0.256
ClinicalBERT	Syn	NL	section	0.044	0.067	0.076	0.082	0.044	0.108	0.174	0.264	0.426	0.592
BioLORD-2023	MTS	KW	full	0.462	0.568	0.581	0.584	0.462	0.734	0.830	0.882	0.946	0.976
BioLORD-2023	MTS	NL	full	0.688	0.762	0.768	0.770	0.688	0.874	0.924	0.944	0.980	0.988
BioLORD-2023	MTS	KW	f256	0.454	0.547	0.561	0.565	0.454	0.710	0.808	0.876	0.942	0.964
BioLORD-2023	MTS	NL	f256	0.760	0.808	0.814	0.816	0.760	0.884	0.928	0.954	0.978	0.988
BioLORD-2023	MTS	KW	f512	0.442	0.552	0.564	0.570	0.442	0.716	0.806	0.884	0.940	0.974
BioLORD-2023	MTS	NL	f512	0.696	0.770	0.775	0.777	0.696	0.884	0.922	0.954	0.982	0.988
BioLORD-2023	MTS	KW	section	0.462	0.568	0.581	0.584	0.462	0.734	0.830	0.882	0.946	0.976
BioLORD-2023	MTS	NL	section	0.688	0.762	0.768	0.770	0.688	0.874	0.924	0.944	0.980	0.988
BioLORD-2023	PMC	KW	full	0.154	0.210	0.225	0.233	0.154	0.304	0.416	0.528	0.690	0.788
BioLORD-2023	PMC	NL	full	0.842	0.883	0.884	0.886	0.842	0.950	0.964	0.984	0.996	0.998
BioLORD-2023	PMC	KW	f256	0.130	0.181	0.193	0.199	0.130	0.272	0.358	0.456	0.596	0.706
BioLORD-2023	PMC	NL	f256	0.914	0.936	0.937	0.938	0.914	0.972	0.982	0.990	0.998	0.998
BioLORD-2023	PMC	KW	f512	0.134	0.183	0.193	0.201	0.134	0.264	0.338	0.458	0.602	0.712
BioLORD-2023	PMC	NL	f512	0.840	0.880	0.882	0.883	0.840	0.948	0.962	0.984	0.992	0.998
BioLORD-2023	PMC	KW	section	0.154	0.210	0.225	0.233	0.154	0.304	0.416	0.528	0.692	0.788
BioLORD-2023	PMC	NL	section	0.842	0.883	0.884	0.886	0.842	0.950	0.964	0.984	0.996	0.998
BioLORD-2023	Syn	KW	full	0.066	0.140	0.162	0.182	0.066	0.290	0.456	0.744	0.888	0.940
BioLORD-2023	Syn	NL	full	0.292	0.405	0.419	0.422	0.292	0.588	0.686	0.734	0.804	0.858
BioLORD-2023	Syn	KW	f256	0.080	0.142	0.163	0.178	0.080	0.260	0.418	0.642	0.876	0.946
BioLORD-2023	Syn	NL	f256	0.356	0.462	0.472	0.475	0.356	0.626	0.696	0.748	0.804	0.852
BioLORD-2023	Syn	KW	f512	0.060	0.117	0.137	0.154	0.060	0.234	0.388	0.630	0.878	0.940
BioLORD-2023	Syn	NL	f512	0.272	0.384	0.398	0.401	0.272	0.568	0.668	0.722	0.796	0.840
BioLORD-2023	Syn	KW	section	0.056	0.111	0.133	0.148	0.056	0.230	0.398	0.624	0.862	0.934
BioLORD-2023	Syn	NL	section	0.288	0.394	0.407	0.411	0.288	0.566	0.660	0.720	0.780	0.828
MedCPT	MTS	KW	full	0.514	0.613	0.624	0.628	0.514	0.778	0.856	0.918	0.964	0.984
MedCPT	MTS	NL	full	0.640	0.715	0.725	0.727	0.640	0.836	0.912	0.946	0.984	0.988
MedCPT	MTS	KW	f256	0.532	0.632	0.642	0.645	0.532	0.792	0.868	0.910	0.960	0.976
MedCPT	MTS	NL	f256	0.692	0.757	0.766	0.768	0.692	0.866	0.926	0.952	0.976	0.986
MedCPT	MTS	KW	f512	0.528	0.621	0.630	0.635	0.528	0.776	0.848	0.918	0.956	0.978
MedCPT	MTS	NL	f512	0.636	0.715	0.726	0.728	0.636	0.836	0.918	0.948	0.986	0.990
MedCPT	MTS	KW	section	0.514	0.613	0.624	0.628	0.514	0.778	0.856	0.918	0.964	0.984
MedCPT	MTS	NL	section	0.640	0.715	0.725	0.727	0.640	0.836	0.912	0.946	0.984	0.988
MedCPT	PMC	KW	full	0.490	0.557	0.569	0.573	0.490	0.664	0.752	0.816	0.918	0.960
MedCPT	PMC	NL	full	0.632	0.712	0.720	0.723	0.632	0.838	0.898	0.946	0.978	0.994
MedCPT	PMC	KW	f256	0.486	0.555	0.568	0.573	0.486	0.672	0.766	0.836	0.888	0.950
MedCPT	PMC	NL	f256	0.738	0.804	0.810	0.811	0.738	0.904	0.946	0.968	0.984	0.998
MedCPT	PMC	KW	f512	0.444	0.527	0.536	0.540	0.444	0.658	0.728	0.788	0.896	0.944
MedCPT	PMC	NL	f512	0.622	0.705	0.713	0.716	0.622	0.836	0.892	0.932	0.976	0.992
MedCPT	PMC	KW	section	0.490	0.557	0.569	0.573	0.490	0.664	0.752	0.816	0.918	0.958
MedCPT	PMC	NL	section	0.632	0.711	0.720	0.723	0.632	0.836	0.898	0.946	0.978	0.994
MedCPT	Syn	KW	full	0.100	0.188	0.212	0.225	0.100	0.362	0.550	0.748	0.924	0.972
MedCPT	Syn	NL	full	0.114	0.182	0.201	0.212	0.114	0.312	0.460	0.622	0.772	0.862

Model	Corpus	Query	Chunk	MRR@1	MRR@5	MRR@10	MRR@20	R@1	R@5	R@10	R@20	R@50	R@100
MedCPT	Syn	KW	f256	0.088	0.173	0.196	0.209	0.088	0.338	0.514	0.696	0.864	0.960
MedCPT	Syn	NL	f256	0.102	0.170	0.189	0.200	0.102	0.294	0.432	0.586	0.758	0.826
MedCPT	Syn	KW	f512	0.098	0.185	0.210	0.223	0.098	0.358	0.548	0.744	0.924	0.972
MedCPT	Syn	NL	f512	0.116	0.180	0.200	0.211	0.116	0.304	0.454	0.614	0.768	0.854
MedCPT	Syn	KW	section	0.102	0.189	0.214	0.227	0.102	0.360	0.552	0.746	0.924	0.972
MedCPT	Syn	NL	section	0.116	0.183	0.202	0.213	0.116	0.310	0.458	0.622	0.774	0.860
BGE-base	MTS	KW	full	0.684	0.752	0.759	0.761	0.684	0.854	0.906	0.936	0.964	0.974
BGE-base	MTS	NL	full	0.836	0.880	0.882	0.883	0.836	0.944	0.964	0.978	0.986	0.990
BGE-base	MTS	KW	f256	0.642	0.731	0.735	0.738	0.642	0.870	0.900	0.936	0.970	0.984
BGE-base	MTS	NL	f256	0.846	0.886	0.888	0.889	0.846	0.952	0.968	0.974	0.988	0.990
BGE-base	MTS	KW	f512	0.686	0.754	0.760	0.763	0.686	0.860	0.906	0.946	0.974	0.982
BGE-base	MTS	NL	f512	0.844	0.886	0.888	0.890	0.844	0.948	0.962	0.980	0.990	0.994
BGE-base	MTS	KW	section	0.684	0.752	0.759	0.761	0.684	0.854	0.906	0.936	0.964	0.974
BGE-base	MTS	NL	section	0.836	0.880	0.882	0.883	0.836	0.944	0.964	0.978	0.986	0.990
BGE-base	PMC	KW	full	0.380	0.446	0.459	0.464	0.380	0.558	0.658	0.736	0.846	0.912
BGE-base	PMC	NL	full	0.844	0.880	0.883	0.884	0.844	0.938	0.962	0.976	0.994	0.996
BGE-base	PMC	KW	f256	0.366	0.433	0.441	0.445	0.366	0.554	0.610	0.666	0.772	0.850
BGE-base	PMC	NL	f256	0.858	0.894	0.896	0.897	0.858	0.950	0.966	0.980	0.992	0.994
BGE-base	PMC	KW	f512	0.308	0.368	0.379	0.385	0.308	0.474	0.550	0.636	0.764	0.832
BGE-base	PMC	NL	f512	0.838	0.874	0.877	0.878	0.838	0.932	0.954	0.974	0.992	0.994
BGE-base	PMC	KW	section	0.380	0.446	0.459	0.464	0.380	0.560	0.658	0.734	0.846	0.912
BGE-base	PMC	NL	section	0.844	0.880	0.883	0.884	0.844	0.938	0.962	0.976	0.994	0.996
BGE-base	Syn	KW	full	0.138	0.228	0.253	0.268	0.138	0.398	0.582	0.806	0.942	0.974
BGE-base	Syn	NL	full	0.376	0.482	0.494	0.496	0.376	0.644	0.728	0.762	0.814	0.846
BGE-base	Syn	KW	f256	0.128	0.221	0.245	0.259	0.128	0.404	0.582	0.782	0.920	0.972
BGE-base	Syn	NL	f256	0.370	0.466	0.479	0.482	0.370	0.620	0.708	0.756	0.782	0.830
BGE-base	Syn	KW	f512	0.122	0.211	0.236	0.250	0.122	0.380	0.566	0.774	0.922	0.970
BGE-base	Syn	NL	f512	0.376	0.475	0.486	0.490	0.376	0.632	0.716	0.760	0.806	0.836
BGE-base	Syn	KW	section	0.124	0.214	0.238	0.252	0.124	0.388	0.572	0.774	0.930	0.978
BGE-base	Syn	NL	section	0.372	0.463	0.477	0.480	0.372	0.612	0.706	0.752	0.794	0.826
GTE-base	MTS	KW	full	0.640	0.721	0.730	0.732	0.640	0.844	0.904	0.938	0.958	0.978
GTE-base	MTS	NL	full	0.840	0.883	0.885	0.886	0.840	0.948	0.966	0.976	0.988	0.992
GTE-base	MTS	KW	f256	0.642	0.717	0.724	0.727	0.642	0.836	0.888	0.932	0.960	0.976
GTE-base	MTS	NL	f256	0.864	0.899	0.901	0.901	0.864	0.954	0.970	0.976	0.988	0.992
GTE-base	MTS	KW	f512	0.626	0.712	0.721	0.723	0.626	0.844	0.914	0.946	0.970	0.976
GTE-base	MTS	NL	f512	0.850	0.889	0.892	0.892	0.850	0.952	0.970	0.978	0.992	0.994
GTE-base	MTS	KW	section	0.640	0.721	0.730	0.732	0.640	0.844	0.904	0.938	0.958	0.978
GTE-base	MTS	NL	section	0.840	0.883	0.885	0.886	0.840	0.948	0.966	0.976	0.988	0.992
GTE-base	PMC	KW	full	0.326	0.400	0.413	0.419	0.326	0.522	0.614	0.708	0.804	0.888
GTE-base	PMC	NL	full	0.858	0.898	0.902	0.902	0.858	0.952	0.978	0.990	0.994	0.998
GTE-base	PMC	KW	f256	0.298	0.346	0.358	0.365	0.298	0.436	0.522	0.632	0.734	0.810
GTE-base	PMC	NL	f256	0.868	0.894	0.897	0.898	0.868	0.944	0.966	0.976	0.992	0.996
GTE-base	PMC	KW	f512	0.242	0.295	0.307	0.314	0.242	0.384	0.476	0.574	0.708	0.794
GTE-base	PMC	NL	f512	0.846	0.885	0.889	0.890	0.846	0.942	0.970	0.984	0.990	0.992
GTE-base	PMC	KW	section	0.326	0.400	0.413	0.419	0.326	0.522	0.614	0.708	0.804	0.888
GTE-base	PMC	NL	section	0.858	0.898	0.902	0.902	0.858	0.952	0.978	0.990	0.994	0.998

Model	Corpus	Query	Chunk	MRR@1	MRR@5	MRR@10	MRR@20	R@1	R@5	R@10	R@20	R@50	R@100
GTE-base	Syn	KW	full	0.118	0.200	0.219	0.236	0.118	0.370	0.518	0.772	0.936	0.970
GTE-base	Syn	NL	full	0.356	0.460	0.469	0.473	0.356	0.634	0.698	0.752	0.780	0.818
GTE-base	Syn	KW	f256	0.100	0.175	0.198	0.211	0.100	0.328	0.504	0.690	0.906	0.956
GTE-base	Syn	NL	f256	0.336	0.441	0.451	0.453	0.336	0.602	0.674	0.710	0.752	0.782
GTE-base	Syn	KW	f512	0.096	0.172	0.192	0.207	0.096	0.320	0.468	0.688	0.870	0.940
GTE-base	Syn	NL	f512	0.346	0.438	0.447	0.450	0.346	0.598	0.668	0.710	0.738	0.770
GTE-base	Syn	KW	section	0.102	0.177	0.199	0.213	0.102	0.318	0.488	0.706	0.878	0.934
GTE-base	Syn	NL	section	0.350	0.435	0.448	0.451	0.350	0.572	0.666	0.708	0.738	0.764
Nomic-embed-t.	MTS	KW	full	0.704	0.763	0.768	0.771	0.704	0.858	0.894	0.926	0.960	0.974
Nomic-embed-t.	MTS	NL	full	0.850	0.886	0.889	0.890	0.850	0.940	0.960	0.972	0.986	0.988
Nomic-embed-t.	MTS	KW	f256	0.676	0.739	0.745	0.748	0.676	0.840	0.882	0.926	0.948	0.962
Nomic-embed-t.	MTS	NL	f256	0.850	0.889	0.891	0.891	0.850	0.942	0.952	0.962	0.974	0.982
Nomic-embed-t.	MTS	KW	f512	0.690	0.749	0.754	0.757	0.690	0.846	0.880	0.918	0.956	0.974
Nomic-embed-t.	MTS	NL	f512	0.852	0.886	0.889	0.890	0.852	0.930	0.956	0.970	0.978	0.988
Nomic-embed-t.	MTS	KW	section	0.704	0.763	0.768	0.771	0.704	0.858	0.894	0.926	0.960	0.974
Nomic-embed-t.	MTS	NL	section	0.850	0.886	0.889	0.890	0.850	0.940	0.960	0.972	0.986	0.988
Nomic-embed-t.	PMC	KW	full	0.386	0.450	0.460	0.465	0.386	0.562	0.632	0.702	0.806	0.856
Nomic-embed-t.	PMC	NL	full	0.926	0.949	0.950	0.950	0.926	0.980	0.986	0.990	0.996	0.998
Nomic-embed-t.	PMC	KW	f256	0.392	0.451	0.460	0.464	0.392	0.552	0.626	0.680	0.754	0.832
Nomic-embed-t.	PMC	NL	f256	0.954	0.966	0.967	0.967	0.954	0.986	0.990	0.992	0.994	1.000
Nomic-embed-t.	PMC	KW	f512	0.332	0.396	0.406	0.410	0.332	0.502	0.574	0.642	0.724	0.800
Nomic-embed-t.	PMC	NL	f512	0.914	0.942	0.943	0.943	0.914	0.980	0.988	0.990	0.996	0.998
Nomic-embed-t.	PMC	KW	section	0.388	0.451	0.461	0.465	0.388	0.562	0.632	0.702	0.806	0.856
Nomic-embed-t.	PMC	NL	section	0.926	0.949	0.950	0.950	0.926	0.980	0.986	0.990	0.996	0.998
Nomic-embed-t.	Syn	KW	full	0.168	0.265	0.288	0.302	0.168	0.446	0.620	0.822	0.944	0.978
Nomic-embed-t.	Syn	NL	full	0.408	0.500	0.510	0.512	0.408	0.650	0.724	0.764	0.822	0.888
Nomic-embed-t.	Syn	KW	f256	0.146	0.234	0.258	0.270	0.146	0.404	0.582	0.762	0.900	0.960
Nomic-embed-t.	Syn	NL	f256	0.392	0.487	0.498	0.501	0.392	0.636	0.710	0.756	0.834	0.896
Nomic-embed-t.	Syn	KW	f512	0.166	0.261	0.283	0.297	0.166	0.434	0.606	0.802	0.940	0.972
Nomic-embed-t.	Syn	NL	f512	0.406	0.497	0.507	0.510	0.406	0.648	0.718	0.760	0.820	0.888
Nomic-embed-t.	Syn	KW	section	0.152	0.249	0.270	0.284	0.152	0.426	0.588	0.782	0.938	0.976
Nomic-embed-t.	Syn	NL	section	0.388	0.486	0.496	0.499	0.388	0.646	0.718	0.762	0.820	0.882
OpenAI-emb3-s.	MTS	KW	full	0.598	0.705	0.711	0.713	0.598	0.876	0.918	0.946	0.974	0.992
OpenAI-emb3-s.	MTS	NL	full	0.792	0.859	0.862	0.863	0.792	0.950	0.968	0.986	0.992	0.996
OpenAI-emb3-s.	MTS	KW	f256	0.648	0.735	0.742	0.743	0.648	0.872	0.916	0.932	0.962	0.976
OpenAI-emb3-s.	MTS	NL	f256	0.836	0.888	0.890	0.891	0.836	0.954	0.964	0.974	0.986	0.994
OpenAI-emb3-s.	MTS	KW	f512	0.630	0.715	0.723	0.725	0.630	0.860	0.912	0.942	0.962	0.986
OpenAI-emb3-s.	MTS	NL	f512	0.814	0.873	0.874	0.875	0.814	0.952	0.962	0.978	0.992	0.994
OpenAI-emb3-s.	MTS	KW	section	0.598	0.705	0.710	0.713	0.598	0.876	0.918	0.946	0.974	0.992
OpenAI-emb3-s.	MTS	NL	section	0.790	0.858	0.860	0.862	0.790	0.950	0.968	0.986	0.992	0.996
OpenAI-emb3-s.	PMC	KW	full	0.326	0.397	0.410	0.417	0.326	0.520	0.612	0.720	0.808	0.888

Model	Corpus	Query	Chunk	MRR@1	MRR@5	MRR@10	MRR@20	R@1	R@5	R@10	R@20	R@50	R@100
OpenAI-emb3-s.	PMC	NL	full	0.774	0.825	0.829	0.831	0.774	0.906	0.932	0.964	0.982	0.992
OpenAI-emb3-s.	PMC	KW	f256	0.398	0.470	0.481	0.488	0.398	0.586	0.664	0.756	0.846	0.894
OpenAI-emb3-s.	PMC	NL	f256	0.982	0.987	0.987	0.988	0.982	0.994	0.996	1.000	1.000	1.000
OpenAI-emb3-s.	PMC	KW	f512	0.342	0.422	0.433	0.441	0.342	0.558	0.636	0.738	0.830	0.898
OpenAI-emb3-s.	PMC	NL	f512	0.912	0.941	0.942	0.943	0.912	0.980	0.988	0.994	0.998	1.000
OpenAI-emb3-s.	PMC	KW	section	0.324	0.396	0.409	0.416	0.324	0.520	0.614	0.716	0.808	0.888
OpenAI-emb3-s.	PMC	NL	section	0.774	0.825	0.828	0.831	0.774	0.906	0.932	0.964	0.982	0.992
OpenAI-emb3-s.	Syn	KW	full	0.138	0.245	0.273	0.289	0.138	0.438	0.650	0.886	0.974	0.990
OpenAI-emb3-s.	Syn	NL	full	0.336	0.446	0.454	0.459	0.336	0.618	0.682	0.748	0.794	0.860
OpenAI-emb3-s.	Syn	KW	f256	0.150	0.245	0.272	0.289	0.150	0.436	0.636	0.874	0.970	0.988
OpenAI-emb3-s.	Syn	NL	f256	0.390	0.485	0.495	0.498	0.390	0.646	0.714	0.750	0.804	0.858
OpenAI-emb3-s.	Syn	KW	f512	0.120	0.217	0.244	0.259	0.120	0.402	0.606	0.830	0.968	0.988
OpenAI-emb3-s.	Syn	NL	f512	0.298	0.400	0.412	0.417	0.298	0.580	0.678	0.734	0.784	0.846
OpenAI-emb3-s.	Syn	KW	section	0.154	0.257	0.282	0.298	0.154	0.454	0.652	0.874	0.978	0.990
OpenAI-emb3-s.	Syn	NL	section	0.338	0.444	0.454	0.458	0.338	0.604	0.678	0.738	0.780	0.842
E5-Mistral-7B	MTS	KW	full	0.032	0.056	0.062	0.066	0.032	0.096	0.142	0.204	0.316	0.510
E5-Mistral-7B	MTS	NL	full	0.106	0.148	0.158	0.163	0.106	0.226	0.302	0.372	0.500	0.634
E5-Mistral-7B	MTS	KW	f256	0.086	0.128	0.139	0.145	0.086	0.202	0.286	0.368	0.512	0.602
E5-Mistral-7B	MTS	NL	f256	0.180	0.240	0.252	0.257	0.180	0.348	0.434	0.514	0.648	0.738
E5-Mistral-7B	MTS	KW	f512	0.052	0.081	0.089	0.093	0.052	0.134	0.196	0.262	0.392	0.500
E5-Mistral-7B	MTS	NL	f512	0.162	0.219	0.228	0.232	0.162	0.330	0.388	0.458	0.594	0.682
E5-Mistral-7B	MTS	KW	section	0.032	0.056	0.062	0.066	0.032	0.096	0.142	0.204	0.316	0.510
E5-Mistral-7B	MTS	NL	section	0.106	0.148	0.158	0.163	0.106	0.226	0.302	0.372	0.500	0.634
E5-Mistral-7B	PMC	KW	full	0.112	0.160	0.169	0.173	0.112	0.248	0.314	0.378	0.526	0.646
E5-Mistral-7B	PMC	NL	full	0.098	0.210	0.226	0.232	0.098	0.382	0.500	0.594	0.736	0.830
E5-Mistral-7B	PMC	KW	f256	0.074	0.108	0.120	0.126	0.074	0.170	0.256	0.340	0.436	0.554
E5-Mistral-7B	PMC	NL	f256	0.030	0.049	0.054	0.059	0.030	0.082	0.120	0.192	0.326	0.470
E5-Mistral-7B	PMC	KW	f512	0.074	0.111	0.121	0.126	0.074	0.174	0.242	0.322	0.430	0.542
E5-Mistral-7B	PMC	NL	f512	0.044	0.057	0.064	0.068	0.044	0.088	0.140	0.212	0.418	0.624
E5-Mistral-7B	PMC	KW	section	0.112	0.160	0.169	0.173	0.112	0.248	0.314	0.378	0.526	0.646
E5-Mistral-7B	PMC	NL	section	0.098	0.210	0.226	0.232	0.098	0.382	0.500	0.594	0.736	0.830
E5-Mistral-7B	Syn	KW	full	0.020	0.034	0.042	0.044	0.020	0.066	0.120	0.152	0.218	0.308
E5-Mistral-7B	Syn	NL	full	0.022	0.036	0.041	0.043	0.022	0.062	0.100	0.136	0.210	0.286
E5-Mistral-7B	Syn	KW	f256	0.012	0.022	0.027	0.031	0.012	0.044	0.080	0.130	0.250	0.362
E5-Mistral-7B	Syn	NL	f256	0.022	0.039	0.043	0.045	0.022	0.070	0.094	0.126	0.210	0.314
E5-Mistral-7B	Syn	KW	f512	0.020	0.035	0.040	0.044	0.020	0.060	0.096	0.154	0.242	0.366
E5-Mistral-7B	Syn	NL	f512	0.022	0.036	0.041	0.044	0.022	0.064	0.106	0.152	0.230	0.342
E5-Mistral-7B	Syn	KW	section	0.016	0.042	0.049	0.057	0.016	0.090	0.144	0.260	0.442	0.626
E5-Mistral-7B	Syn	NL	section	0.030	0.059	0.073	0.078	0.030	0.120	0.218	0.288	0.420	0.532

Model	Corpus	Query	Chunk	MRR@1	MRR@5	MRR@10	MRR@20	R@1	R@5	R@10	R@20	R@50	R@100
Phi-3-mini	MTS	KW	full	0.098	0.158	0.175	0.186	0.098	0.266	0.394	0.550	0.708	0.818
Phi-3-mini	MTS	NL	full	0.406	0.520	0.532	0.537	0.406	0.694	0.786	0.856	0.902	0.928
Phi-3-mini	MTS	KW	f256	0.110	0.176	0.189	0.196	0.110	0.296	0.398	0.500	0.662	0.740
Phi-3-mini	MTS	NL	f256	0.436	0.527	0.538	0.543	0.436	0.670	0.754	0.830	0.888	0.912
Phi-3-mini	MTS	KW	f512	0.108	0.177	0.189	0.197	0.108	0.302	0.392	0.510	0.664	0.760
Phi-3-mini	MTS	NL	f512	0.458	0.539	0.549	0.554	0.458	0.684	0.756	0.828	0.882	0.918
Phi-3-mini	MTS	KW	section	0.098	0.158	0.175	0.186	0.098	0.266	0.394	0.550	0.708	0.818
Phi-3-mini	MTS	NL	section	0.406	0.520	0.532	0.537	0.406	0.694	0.786	0.856	0.902	0.928
Phi-3-mini	PMC	KW	full	0.084	0.129	0.140	0.146	0.084	0.208	0.284	0.376	0.496	0.644
Phi-3-mini	PMC	NL	full	0.498	0.570	0.578	0.583	0.498	0.692	0.754	0.816	0.898	0.940
Phi-3-mini	PMC	KW	f256	0.066	0.101	0.108	0.115	0.066	0.168	0.222	0.316	0.412	0.520
Phi-3-mini	PMC	NL	f256	0.796	0.832	0.834	0.836	0.796	0.888	0.910	0.930	0.948	0.968
Phi-3-mini	PMC	KW	f512	0.060	0.086	0.097	0.102	0.060	0.138	0.218	0.296	0.434	0.556
Phi-3-mini	PMC	NL	f512	0.594	0.660	0.668	0.671	0.594	0.770	0.828	0.872	0.918	0.944
Phi-3-mini	PMC	KW	section	0.084	0.129	0.140	0.146	0.084	0.208	0.286	0.376	0.496	0.644
Phi-3-mini	PMC	NL	section	0.498	0.571	0.579	0.583	0.498	0.694	0.756	0.816	0.898	0.940
Phi-3-mini	Syn	KW	full	0.010	0.028	0.031	0.036	0.010	0.066	0.092	0.164	0.298	0.462
Phi-3-mini	Syn	NL	full	0.070	0.110	0.123	0.131	0.070	0.190	0.288	0.404	0.574	0.736
Phi-3-mini	Syn	KW	f256	0.014	0.027	0.031	0.035	0.014	0.048	0.080	0.134	0.240	0.384
Phi-3-mini	Syn	NL	f256	0.086	0.133	0.145	0.154	0.086	0.230	0.318	0.446	0.596	0.712
Phi-3-mini	Syn	KW	f512	0.014	0.027	0.030	0.034	0.014	0.048	0.072	0.132	0.244	0.398
Phi-3-mini	Syn	NL	f512	0.056	0.104	0.114	0.121	0.056	0.196	0.268	0.372	0.570	0.716
Phi-3-mini	Syn	KW	section	0.012	0.022	0.028	0.031	0.012	0.044	0.088	0.142	0.244	0.394
Phi-3-mini	Syn	NL	section	0.064	0.100	0.114	0.122	0.064	0.170	0.274	0.386	0.544	0.706
E5-Mistral-7B.	MTS	KW	full	0.192	0.328	0.344	0.352	0.192	0.554	0.670	0.782	0.868	0.920
E5-Mistral-7B.	MTS	NL	full	0.568	0.669	0.677	0.680	0.568	0.812	0.872	0.910	0.950	0.972
E5-Mistral-7B.	MTS	KW	f256	0.166	0.261	0.278	0.286	0.166	0.422	0.552	0.672	0.814	0.878
E5-Mistral-7B.	MTS	NL	f256	0.522	0.621	0.630	0.633	0.522	0.780	0.844	0.888	0.918	0.948
E5-Mistral-7B.	MTS	KW	f512	0.158	0.253	0.273	0.281	0.158	0.430	0.580	0.704	0.834	0.898
E5-Mistral-7B.	MTS	NL	f512	0.542	0.636	0.643	0.647	0.542	0.778	0.826	0.884	0.930	0.952
E5-Mistral-7B.	MTS	KW	section	0.192	0.328	0.344	0.352	0.192	0.554	0.670	0.782	0.868	0.920
E5-Mistral-7B.	MTS	NL	section	0.568	0.669	0.677	0.680	0.568	0.812	0.872	0.910	0.950	0.972
E5-Mistral-7B.	PMC	KW	full	0.138	0.186	0.195	0.202	0.138	0.272	0.340	0.436	0.568	0.718
E5-Mistral-7B.	PMC	NL	full	0.570	0.630	0.638	0.642	0.570	0.728	0.786	0.850	0.912	0.942
E5-Mistral-7B.	PMC	KW	f256	0.076	0.119	0.127	0.134	0.076	0.196	0.262	0.354	0.486	0.610
E5-Mistral-7B.	PMC	NL	f256	0.854	0.878	0.881	0.883	0.854	0.920	0.942	0.960	0.978	0.988
E5-Mistral-7B.	PMC	KW	f512	0.048	0.079	0.089	0.095	0.048	0.130	0.204	0.286	0.440	0.586
E5-Mistral-7B.	PMC	NL	f512	0.674	0.738	0.742	0.745	0.674	0.832	0.864	0.910	0.942	0.958
E5-Mistral-7B.	PMC	KW	section	0.138	0.185	0.195	0.201	0.138	0.272	0.340	0.434	0.568	0.718
E5-Mistral-7B.	PMC	NL	section	0.570	0.631	0.638	0.642	0.570	0.730	0.788	0.850	0.912	0.942
E5-Mistral-7B.	Syn	KW	full	0.066	0.128	0.152	0.167	0.066	0.254	0.432	0.662	0.872	0.938
E5-Mistral-7B.	Syn	NL	full	0.198	0.289	0.305	0.313	0.198	0.444	0.560	0.676	0.766	0.860
E5-Mistral-7B.	Syn	KW	f256	0.048	0.087	0.105	0.115	0.048	0.176	0.306	0.452	0.662	0.774
E5-Mistral-7B.	Syn	NL	f256	0.220	0.302	0.320	0.326	0.220	0.450	0.580	0.670	0.712	0.762
E5-Mistral-7B.	Syn	KW	f512	0.046	0.102	0.124	0.139	0.046	0.210	0.380	0.588	0.818	0.916
E5-Mistral-7B.	Syn	NL	f512	0.162	0.246	0.265	0.272	0.162	0.394	0.532	0.642	0.746	0.838
E5-Mistral-7B.	Syn	KW	section	0.038	0.088	0.107	0.121	0.038	0.192	0.340	0.538	0.786	0.894

Model	Corpus	Query	Chunk	MRR@1	MRR@5	MRR@10	MRR@20	R@1	R@5	R@10	R@20	R@50	R@100
E5-Mistral-7B.	Syn	NL	section	0.142	0.233	0.251	0.259	0.142	0.386	0.524	0.642	0.736	0.832
Nomic-embed-t.	MTS	KW	full	0.674	0.739	0.746	0.747	0.674	0.844	0.888	0.918	0.956	0.978
Nomic-embed-t.	MTS	NL	full	0.846	0.882	0.884	0.885	0.846	0.936	0.954	0.968	0.984	0.988
Nomic-embed-t.	MTS	KW	f256	0.632	0.703	0.709	0.712	0.632	0.808	0.854	0.902	0.948	0.964
Nomic-embed-t.	MTS	NL	f256	0.854	0.888	0.890	0.891	0.854	0.940	0.950	0.968	0.978	0.986
Nomic-embed-t.	MTS	KW	f512	0.650	0.717	0.724	0.726	0.650	0.830	0.878	0.910	0.948	0.972
Nomic-embed-t.	MTS	NL	f512	0.862	0.893	0.895	0.896	0.862	0.936	0.952	0.968	0.978	0.992
Nomic-embed-t.	MTS	KW	section	0.674	0.739	0.746	0.747	0.674	0.844	0.888	0.918	0.956	0.978
Nomic-embed-t.	MTS	NL	section	0.846	0.882	0.884	0.885	0.846	0.936	0.954	0.968	0.984	0.988
Nomic-embed-t.	PMC	KW	full	0.316	0.390	0.399	0.406	0.316	0.526	0.598	0.698	0.786	0.864
Nomic-embed-t.	PMC	NL	full	0.942	0.958	0.959	0.959	0.942	0.982	0.990	0.992	0.996	1.000
Nomic-embed-t.	PMC	KW	f256	0.236	0.305	0.318	0.324	0.236	0.432	0.526	0.612	0.710	0.788
Nomic-embed-t.	PMC	NL	f256	0.942	0.960	0.961	0.961	0.942	0.984	0.990	0.992	0.994	0.998
Nomic-embed-t.	PMC	KW	f512	0.220	0.283	0.293	0.300	0.220	0.398	0.476	0.578	0.688	0.784
Nomic-embed-t.	PMC	NL	f512	0.932	0.953	0.954	0.955	0.932	0.982	0.990	0.994	0.996	1.000
Nomic-embed-t.	PMC	KW	section	0.316	0.390	0.399	0.406	0.316	0.526	0.598	0.700	0.786	0.864
Nomic-embed-t.	PMC	NL	section	0.942	0.958	0.959	0.959	0.942	0.982	0.990	0.992	0.996	1.000
Nomic-embed-t.	Syn	KW	full	0.192	0.291	0.314	0.327	0.192	0.482	0.658	0.846	0.962	0.982
Nomic-embed-t.	Syn	NL	full	0.396	0.496	0.503	0.507	0.396	0.660	0.718	0.768	0.830	0.896
Nomic-embed-t.	Syn	KW	f256	0.168	0.265	0.291	0.303	0.168	0.442	0.634	0.808	0.934	0.978
Nomic-embed-t.	Syn	NL	f256	0.406	0.505	0.512	0.517	0.406	0.658	0.710	0.772	0.826	0.888
Nomic-embed-t.	Syn	KW	f512	0.174	0.279	0.301	0.313	0.174	0.478	0.642	0.824	0.964	0.982
Nomic-embed-t.	Syn	NL	f512	0.396	0.494	0.502	0.505	0.396	0.658	0.712	0.766	0.824	0.896
Nomic-embed-t.	Syn	KW	section	0.172	0.277	0.298	0.311	0.172	0.484	0.636	0.820	0.960	0.982
Nomic-embed-t.	Syn	NL	section	0.386	0.488	0.497	0.501	0.386	0.652	0.720	0.766	0.824	0.890

Abbreviations: MTS, MTSamples; PMC, PMC-Patients; Syn/Synth, Synthetic; KW, keyword; NL, natural language; f256/f512, fixed 256/512-token chunks; sec/section, section-based chunking; R@k, Recall@k; Hit@k, HitRate@k. MRR, Mean Reciprocal Rank; NDCG, Normalized Discounted Cumulative Gain. Full data available as CSV in the replication repository.
